# Rapid generation of *Shigella flexneri* GMMA displaying natural or new and cross-reactive O-Antigens

**DOI:** 10.1038/s41541-022-00497-7

**Published:** 2022-06-30

**Authors:** Gianmarco Gasperini, Maria Michelina Raso, Fabiola Schiavo, Maria Grazia Aruta, Neil Ravenscroft, Barbara Bellich, Paola Cescutti, Francesca Necchi, Rino Rappuoli, Francesca Micoli

**Affiliations:** 1 GSK Vaccines Institute for Global Health (GVGH), Siena, Italy; 2 Università di Trieste, Trieste, Italy; 3 University of Cape Town, Rondebosch, South Africa; 4 GSK Vaccines, Siena, Italy

**Keywords:** Bacteria, Vaccines

## Abstract

Generalized modules for membrane antigens (GMMA) are exosomes released from engineered Gram-negative bacteria and represent an attractive vaccine platform for the delivery of the O-Antigen (OAg), recognized as the key target for protective immunity against several pathogens such as *Shigella*. *Shigella* is a major cause of disease in Low- and Middle-Income countries and the development of a vaccine needs to deal with its large serotypic diversity. All *S. flexneri* serotypes, except serotype 6, share a conserved OAg backbone, corresponding to serotype Y. Here, a GMMA-producing *S. flexneri* scaffold strain displaying the OAg backbone was engineered with different OAg-modifying enzymes, either individually or in combinations. This strategy rapidly yielded GMMA displaying 12 natural serotypes and 16 novel serotypes expressing multiple epitopes combinations that do not occur in nature. Importantly, a candidate GMMA displaying a hybrid OAg elicited broadly cross-bactericidal antibodies against a large panel of *S. flexneri* serotypes.

## Introduction

Gram-negative bacteria spontaneously release exosomes from their outer membrane, also called outer membrane vesicles (OMV), containing surface exposed antigens in their native environment together with immuno-stimulatory molecules, such as lipopolysaccharide (LPS), lipoproteins and peptidoglycans^1–3^. GMMA are OMV derived from bacteria genetically engineered to enhance their natural vesiculation^4,5^. Recently GMMA have been proposed as an innovative vaccine platform for the delivery of the O-Antigen (OAg), known to be the key target for protective immunity against several pathogens^6–8^.

*Shigella* is a major cause of disease with >200,000 deaths annually^9,10^. Almost all deaths occur in developing countries and many of them in children under the age of 5 years. Protective immunity following *Shigella* infection seems to be predominantly directed against the serotype-specific OAg and many OAg-based vaccines are currently in development^11–14^. The *Shigella* genus is divided into 4 species and more than 50 antigenically distinct serotypes^15^. The prevalence of these serotypes varies by socio-economic status within countries and changes over time even within the same geographical region^16–20^. *S. sonnei* is geographically the most widespread and dominates in economically developed countries, *S. flexneri* serotypes are more important in developing countries while *S. boydii* and *S. dysenteriae* occur at much lower frequencies^16,21^. All these factors contribute to the complexity of vaccine development which needs to compromise between the number of components resulting in sufficient coverage and manufacturing affordability for low- and middle-income countries (LMIC)^22^. Two recent prospective studies examined *Shigella* diversity to inform vaccine development: the Multicenter *Shigella* Surveillance (MCSS) study and the Global Enteric Multicenter (GEM) study^16,23^. Both studies highlighted the need to include *S. sonnei* and different *S. flexneri* serotypes in a broadly-protective vaccine.

All serotypes of *S. flexneri*, except serotype 6, share a conserved polysaccharide backbone (corresponding to serotype Y) consisting of the following OAg repeating unit (RU)^15,24^:

$$\left. { \to 2} \right) {\hbox{-}} \upalpha {\hbox{-}} {{{\mathrm{L}}}} {\hbox{-}} {{{\mathrm{Rha}}}}p^{{{{\mathrm{III}}}}} {\hbox{-}} (1 \to 2) {\hbox{-}} \upalpha {\hbox{-}} {{{\mathrm{L}}}} {\hbox{-}} {{{\mathrm{Rha}}}}p^{{{{\mathrm{II}}}}} {\hbox{-}} (1 \to 3) {\hbox{-}} \upalpha {\hbox{-}} {{{\mathrm{L}}}} {\hbox{-}} {{{\mathrm{Rha}}}}p^{{{\mathrm{I}}}} {\hbox{-}} (1 \to 3) {\hbox{-}} \upbeta {\hbox{-}} {{{\mathrm{D}}}} {\hbox{-}} {{{\mathrm{Glc}}p{\rm{NAc}}}} {\hbox{-}} \left( {1 \to } \right..$$

The diversity of *S. flexneri* serotypes is due to the modification of this common OAg backbone with glucosyl and/or O-acetyl groups, as a result of bacteriophages infection and acquisition of OAg-modifying enzymes like glucosyltransferases and O-acetyltransferases^25,26^. The variety of O-acetyl and glucosyl groups on the OAg RU generates all type (I–V) and group (3(4), 6, 7(8), 9, 10) specificities that define the different serotypes, subtypes and subvariants of *S. flexneri*^27^.

In this study, we present a new approach for the rapid generation of GMMA displaying single or multiple OAg from different *Shigella* serotypes. We started by generating a well characterized strain of *S. flexneri* suitable for GMMA production and displaying the serotype Y OAg backbone. Such scaffold strain was engineered with a series of OAg-modifying enzymes, either individually or in combination, to convert serotype Y to all possible natural serotypes. This yielded 12 GMMA-producing strains which differ from each other only in the nature of the displayed OAg. Selected GMMA obtained from these converted strains were compared to GMMA obtained from natural *S. flexneri* strains for their structural features and immunogenicity in mice. Moreover, the scaffold strain and a *S. flexneri* serotype 3a strain were engineered with combinations of OAg-modifying enzymes which naturally do not occur together. This resulted in 16 previously undescribed serotypes displaying multiple type and group factors at the same time and potentially able to induce cross-reactive immune responses.

## Results

### Generation and transformation of a *S. flexneri* scaffold strain

**Fig. 1:**
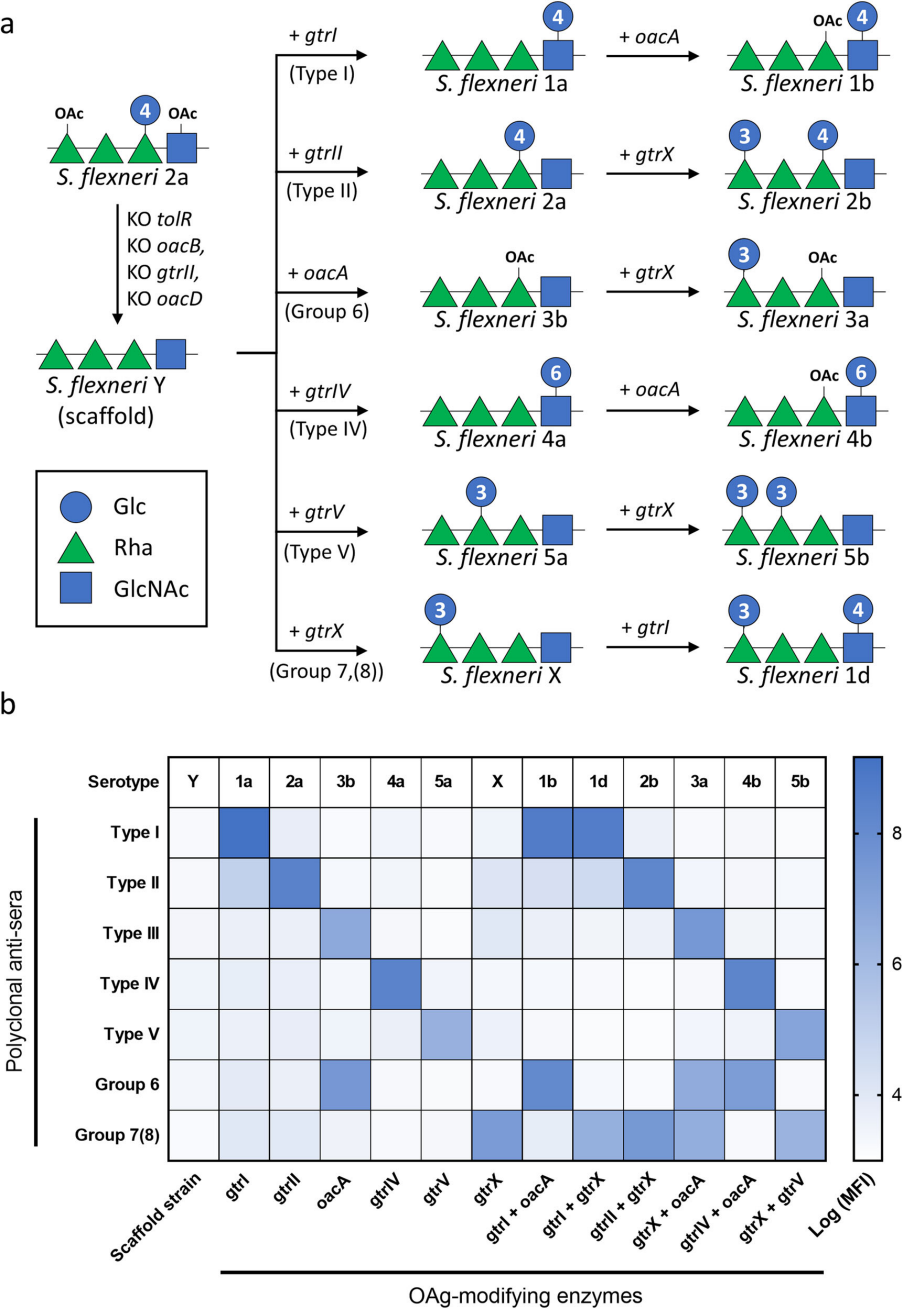
Conversion of serotype Y scaffold strain to different *S. flexneri* serotpes. **a** Engineering strategy and resulting OAg structures as verified by ^1^H-NMR. The numbers inside the blue circles refer to the position of the linkage of glucose to the RU. **b** FACS typing analysis of all strains with Denka Seiken monovalent antisera kit. Results are shown as a heat-map of log-transformed mean fluorescence intensities (MFI).

At first, we generated a GMMA-producing strain by removing the *tolR* gene in *S. flexneri* serotype 2a. Then, we converted *S. flexneri* serotype 2a Δ*tolR* into a serotype Y scaffold strain by removing the phage genes responsible for O-acetylation of Rha^III^ (*oacB* gene), glucosylation of Rha^I^ (*gtrII* gene) and O-acetylation of GlcNAc (*oacD* gene) (Supplementary Table 1, Fig. 1a). GMMA resulting from the scaffold strain were fully characterized with a panel of analytical methods to confirm the display of the expected OAg (Table 1, Supplementary Fig. 1). Next, we generated a set of plasmids based on the pCOLA-Duet and pACYC-Duet systems for the introduction of five known glucosyltransferases (*gtrI*, *gtrII*, *gtrIV*, *gtrV*, *gtrX*) and one O-acetyltransferase (*oacA*) responsible for the majority of the OAg modifications in *S. flexneri* (Supplementary Tables 1 and 2). Transformation of the *S. flexneri* serotype Y scaffold strain with the six plasmids and their combinations yielded 12 converted strains (Fig. 1a, Supplementary Table 2). FACS typing analysis on the bacterial cells and nuclear magnetic resonance (NMR) spectroscopy on the OAg extracted from the corresponding GMMA confirmed all the expected OAg structures resulting from the conversion of serotype Y to all known *S. flexneri* serotypes (1a, 1b, 1d, 2a, 2b, 3a, 3b, 4a, 4b, 5a, 5b, X) (Fig. 1b, Supplementary Fig. 1).

**Table 1:** Analytical characterization of the GMMA used in the immunogenicity studies.

GMMA ID	GMMA from strain	OAg/protein ratio (w/w)	OAg size and peak area % (HPLC-SEC dRI)	Rha/Glc ratio (HPAEC-PAD)
GMMA Y scaffold	*S. flexneri* scaffold Δ*tolR*::*frt*	0.54	77 kDa (16%), 17 kDa (48%), 2 kDa (36%)	NA
GMMA 1a scaffold	*S. flexneri* scaffold Δ*tolR*::*frt* pCOLA-Duet_*gtrI*	0.49	58 kDa (20%), 14 kDa (43%), 2 kDa (37%)	2.4
GMMA 2a scaffold	*S. flexneri* scaffold Δ*tolR*::*frt* pCOLA-Duet_*gtrII*	0.73	59 kDa (22%), 14 kDa (43%), 2 kDa (35%)	2.2
GMMA mixed 1 + 2	*S. flexneri* scaffold Δ*tolR*::*frt* pCOLA-Duet_*gtrI* pACYC-Duet_*gtrII*	0.50	55 kDa (20%), 14 kDa (43%), 2 kDa (37%)	2.4
GMMA hybrid 1 + 3	*S. flexneri* 3a Δ*tolR*::*frt* pCOLA-Duet_*gtrI*	0.65	64 kDa (26%), 16 kDa (46%), 2.5 kDa (28%)	1.5
GMMA 1a natural	*S. flexneri* 1a Δ*tolR*::*aph*	0.60	13 kDa (59%), 2 kDa (41%)	2.6
GMMA 2a natural	*S. flexneri* 2a Δ*tolR*::*frt*	0.40	67 kDa (15%), 14 kDa (50%), 2 kDa (35%)	2.4
GMMA 3a natural	*S. flexneri* 3a Δ*tolR*::*frt*	0.65	71 kDa (22%), 16 kDa (45%), 2.5 kDa (33%)	2.5

### Comparison between GMMA from the converted scaffold strains and GMMA from the corresponding natural strains

**Fig. 2:**
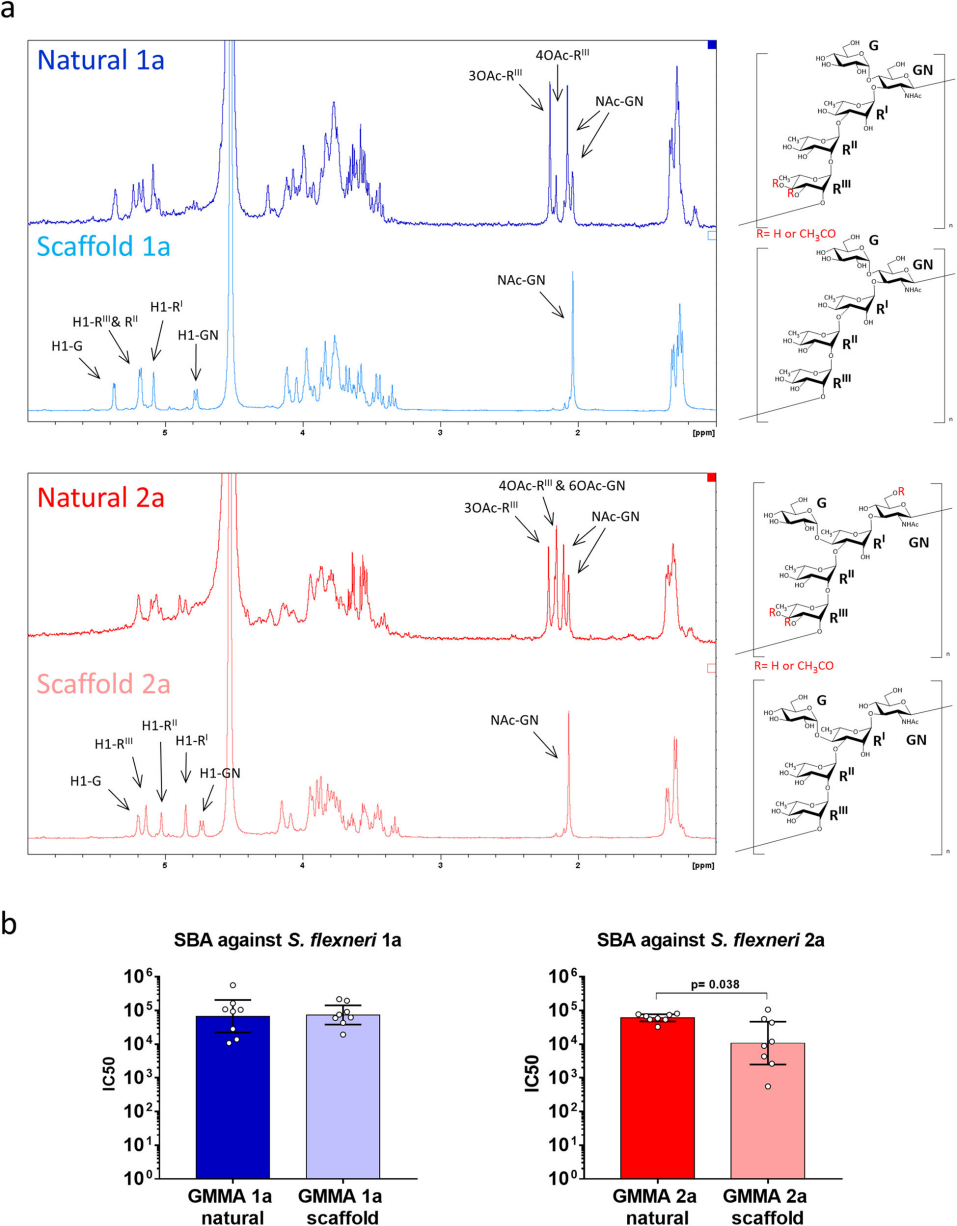
Analytical and immunological comparison between GMMA from the converted scaffold strains and GMMA from the corresponding natural strains. **a** ^1^H-NMR spectra of OAg isolated from GMMA. Chemical shifts were assigned in the anomeric and O-acetyl regions (R = Rhamnose; GN = N-Acetyl Gucosamine; G = Glucose). **b** Bactericidal activity of sera raised in mice against the different GMMA. Serum dilutions able to kill 50% of bacteria in the assay (IC_50_) are reported for individual mice (dots), error bars represent 95% confidence interval.

GMMA from the scaffold strain converted to serotype 1a and 2a were fully characterized and compared to to GMMA from natural *S. flexneri* serotype 1a and 2a (Fig. 2a, Table 1). The few differences observed were related to the presence of O-acetyl groups in GMMA obtained from natural serotypes (OAc-Rha^III^ in serotype 1a, OAc-Rha^III^ and OAc-GlcNAc in serotype 2a) which were not engineered in the scaffold strains (Fig. 2a) and the lack of high molecular mass (HMM) OAg in GMMA obtained from natural serotype 1a (Table 1), most likely dependent on the absence of a specific OAg length regulator. When tested in mice at the same OAg dose, GMMA derived from the converted scaffold strain were able to induce functional antibodies, similarly to the corresponding GMMA derived from natural strains (Fig. 2b). In particular, no difference was observed between natural 1a and scaffold 1a GMMA in the serum bactericidal activity elicited against *S. flexneri* 1a in vitro, while a slightly lower response was measured with scaffold 2a GMMA compared to natural 2a GMMA in the serum bactericidal activity elicited against *S. flexneri* serotype 2a (*p* = 0.038; Mann–Whitney unpaired two-tailed *t*-test) (Fig. 2b).

### Transformation with naturally non-occurring combinations of OAg-modifying enzymes

**Fig. 3:**
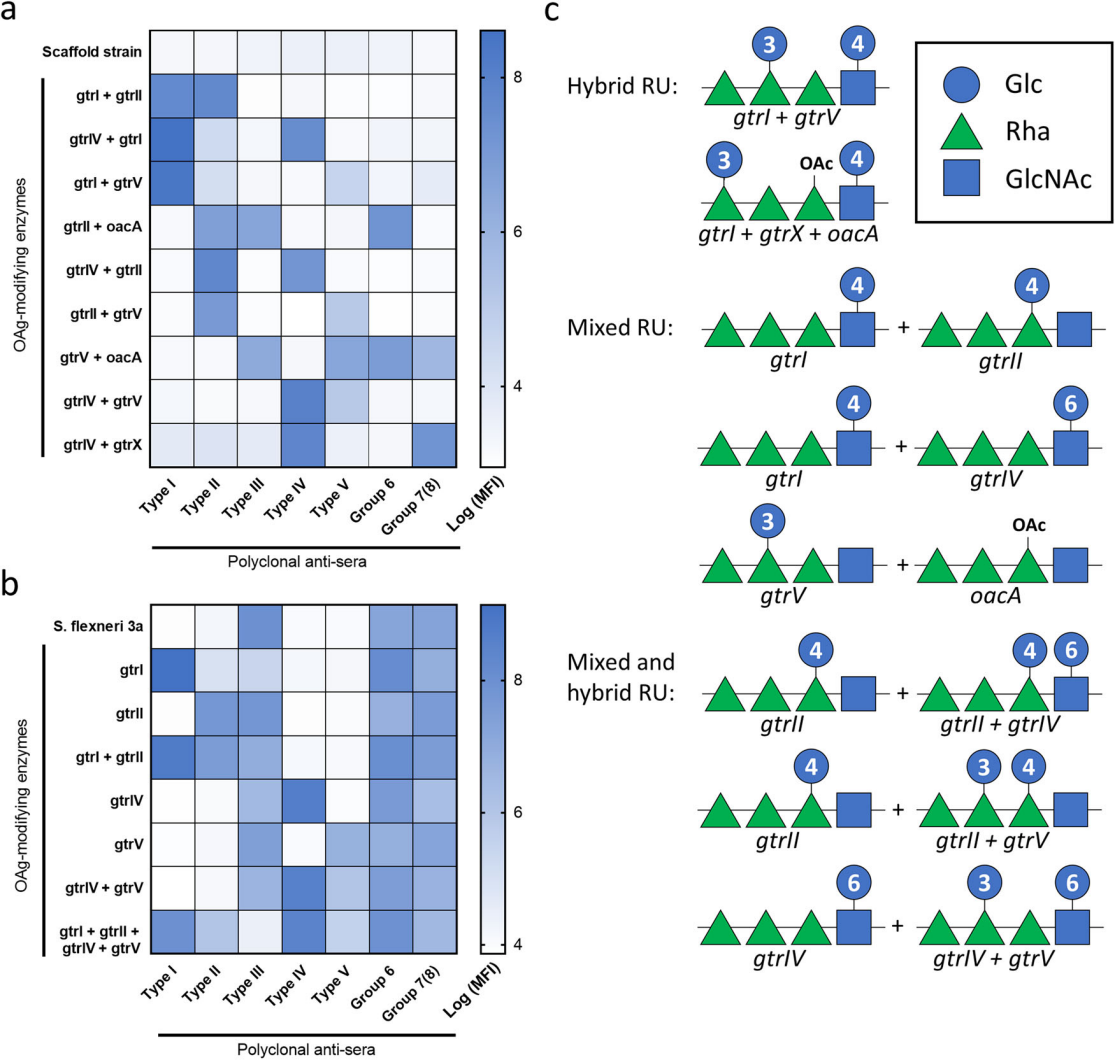
Engineering of naturally non-occurring combinations of OAg-modifying enzymes. **a** FACS typing analysis of all strains generated by transforming the serotype Y scaffold strain. **b** FACS typing analysis of all strains generated by transforming *S. flexneri* 3a. **c** Resulting OAg structures as verified by ^1^H-NMR.

Although certain combinations of OAg-modifying enzymes do not occur in nature due to incompatibility of bacteriophages with respect to infecting the same bacterium^27^, our strategy allowed to engineer *S. flexneri* with naturally non-occurring combinations of glucosyl- and O-acetyl-transferases. Transformation of the scaffold strain with naturally non-occurring combinations of two OAg-modifying enzymes yielded 9 previously undescribed serotypes displaying multiple type and group epitopes simultaneously, as confirmed by FACS typing analysis (Fig. 3a).

To increase the number of co-expressed OAg-modifying enzymes and push the approach even further, we also transformed a *S. flexneri* serotype 3a GMMA-producing strain, which naturally carries the *gtrX* and *oacA* genes (Supplementary Table 1), with the previously described plasmids yielding 7 additional strains displaying up to five type factors and two group factors at the same time (Fig. 3b).

FACS analysis confirmed that all bacterial populations were uniformly expressing the provided enzymes and therefore displaying the corresponding type and group factors (not shown). Nevertheless, it was crucial to understand whether the OAg chains were composed by (a) different RU, each carrying some of the provided type and group factors or (b) identical RU modified by all the provided OAg-modifying enzymes. To discriminate between these two scenarios, the OAg was extracted from GMMA and characterized by use of ^1^H-NMR spectroscopy (Supplementary Fig. 1).

Interestingly, only one combination of two OAg-modifying enzymes (*gtrI* + *gtrV*) and one combination of three OAg-modifying enzymes (*gtrI* + *gtrX* + *oacA*) were able to modify 100% of the RU (“hybrid OAg”, Fig. 3c, Supplementary Fig. 1), similarly to what observed with naturally occurring enzyme combinations. All other combinations of enzymes yielded mixed OAg chains, indicating that certain enzymes are unable to modify the sugar repeat after a previous modification by a different enzyme. In particular, ^1^H-NMR showed that certain combinations of enzymes resulted in OAg chains composed by two different RU corresponding to individual serotypes combined in a 1:1 ratio (i.e. *gtrI* + *gtrII*, *gtrI* + *gtrIV*, *gtrV* + *oacA*) (“mixed OAg”, Fig. 3c, Supplementary Fig. 1). In such cases, anomeric signals characteristic of the individual RU were identified in the mixed OAg spectra and their relative ratio was calculated through integrals (Supplementary Fig. 1). Other combinations resulted in OAg chains composed by two different RU, only one of which was modified by both enzymes (i.e*. gtrII* + *gtrIV*, *gtrII* + *gtrV*, *gtrIV* + *gtrV*) (“mixed and hybrid OAg”, Fig. 3c, Supplementary Fig. 1). In such cases instead, we identified anomeric signals identical to those present in natural RU as well as additional anomeric signals which we attributed to the presence of hybrid RU. Also in this case, the relative ratio of the different RU was calculated through integrals (Supplementary Fig. 1).

### Immunogenicity of GMMA displaying mixed or hybrid serotypes compared to physical mixtures of GMMA displaying the corresponding individual serotypes

**Fig. 4:**
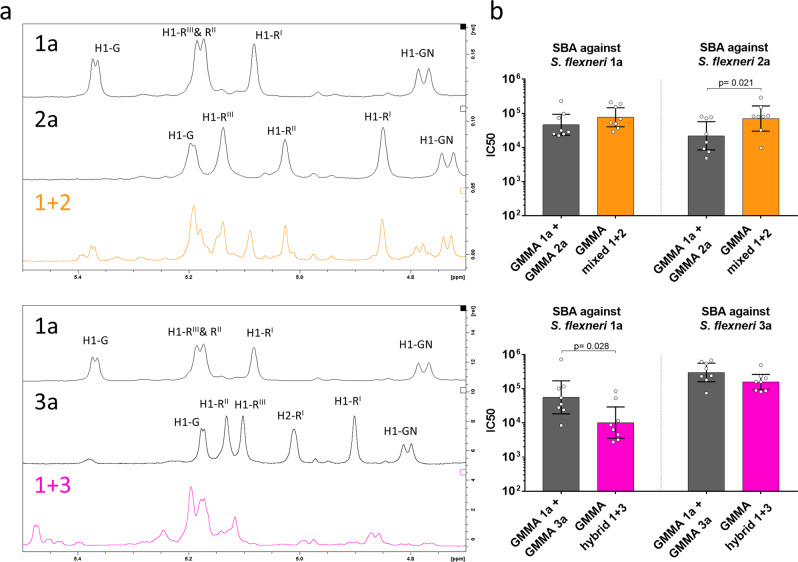
Characterization and immunogenicity of GMMA displaying mixed or hybrid serotypes. **a** Anomeric region of ^1^H-NMR spectra of OAg isolated from GMMA (R = Rhamnose; GN = N-Acetyl Gucosamine; G = Glucose). Mixed or hybrid serotypes were compared to the corresponding individual serotypes. **b** Bactericidal activity of sera raised in mice by GMMA displaying mixed or hybrid serotypes compared to physical mixtures of GMMA displaying the corresponding individual serotypes. Serum dilutions able to kill 50% of bacteria in the assay (IC_50_) are reported for individual mice (dots), error bars represent 95% confidence interval.

Representatives of GMMA displaying mixed or hybrid OAg were selected to investigate their immunogenicity in mice. GMMA from the mixed serotype 1 + 2 (*gtrI* + *gtrII*) and the hybrid serotype 1 + 3 (*gtrI* + *gtrII* + *oacA*) were fully characterized. OAg sugar composition was analyzed using high performance anion exchange chromatography-pulsed amperometric detection (HPAEC-PAD), linkage positions and substitution pattern was determined by use of gas–liquid chromatography–mass spectrometry (GLC-MS) and OAg structure was profiled by ^1^H-NMR spectroscopy (Fig. 4a, Table 1, Supplementary Fig. 1, Supplementary Table 4). GMMA from the mixed serotype 1 + 2 were characterized by OAg chains composed by a mixed population of RU, 50% of which were glucosylated on GlcNAc (as in serotype 1a) and 50% on Rha^I^ (as in serotype 2a). On the other hand, GMMA from the hybrid serotype 1 + 3 were characterized by OAg chains composed by RU glucosylated both on Rha^III^ and on GlcNAc (as in serotype 1d) and O-acetylated on Rha^I^ (similarly to serotype 3a and 3b). Importantly, lot to lot consistency of these structural features was confirmed by characterization of 3 different GMMA preparations at small scale (Supplementary Fig. 2). Both GMMA were tested in mice to compare their immunogenicity to that of physical mixtures of GMMA displaying the corresponding individual serotypes; doses were normalized to administer the same amount of serotype-specific OAg to each group. GMMA carrying mixed or hybrid OAg-induced functional antibodies against both displayed serotypes (Fig. 4b). In particular, GMMA displaying the mixed 1 + 2 serotype elicited higher serum bactericidal activity (SBA) titers against *S. flexneri* 2a than the physical mixture of scaffold 1a and 2a GMMA (*p* = 0.021, Mann–Whitney unpaired two-tailed *t* test). In contrast, GMMA displaying the hybrid 1 + 3 serotype elicited lower SBA titers against *S. flexneri* 1a than the physical mixture of scaffold 1a GMMA and natural 3a GMMA (*p* = 0.028, Mann–Whitney unpaired two-tailed *t* test).

**Fig. 5:**
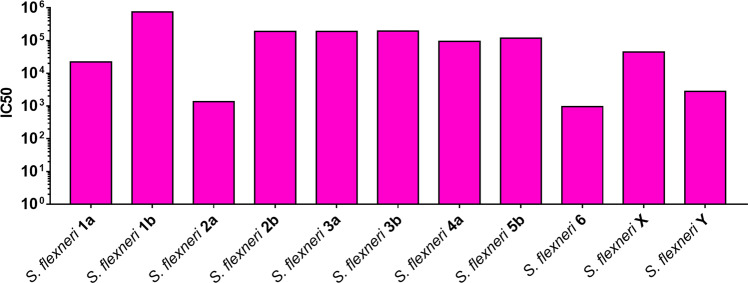
Assessment of cross-reactivity induced by the hybrid 1+3 serotype. Bactericidal activity of sera raised in mice by GMMA displaying hybrid 1+3 serotype against a panel of the most epidemiologically relevant *S. flexneri* serotypes. Pooled sera dilutions able to kill 50% of bacteria in the assay (IC50) are reported.

Based on previously collected cross-reactivity data in mice^28^ and considering the serotype specificities displayed on GMMA from the hybrid 1 + 3 serotype, we tested whether such GMMA could induce broadly cross-reactive antibodies. Importantly the predicted cross-reactivity was observed: SBA analysis of the sera from mice immunized with GMMA obtained from the hybrid 1 + 3 serotype demonstrated their ability to induce killing of all the most epidemiologically relevant *S. flexneri* serotypes (Fig. 5).

## Discussion

A broadly-protective vaccine against shigellosis needs to cover *S. sonnei* and multiple *S. flexneri* serotypes but at the same time needs to balance coverage versus formulation complexity and costs^22^. While *S. sonnei* is characterized by one single serotype, *S. flexneri* consists of a considerable serotype variability, with serotypes 2a, 6, 3a, 2b and 1b being the most frequently isolated in at least two recent multicenter studies^16,23^. *Shigella* vaccines under development span a spectrum of approaches and antigens but almost all *Shigella* vaccines include the OAg, which is considered a protective antigen, although by definition it restricts the vaccine efficacy to homologous or cross-reactive serotypes^12^.

The GMMA technology appears to be a valid platform to develop affordable vaccines^29–32^. Indeed, GMMA can be produced at high yields using a simple and robust manufacturing process^4^. However, GMMA-producing strains are not easily obtained as bacteria need to be genetically engineered to destabilize their outer membrane and increase blebbing^33^. Moreover, mutations are introduced to modify the lipid A structure of the LPS and to minimize the ability of GMMA vaccines to promote reactogenic responses once injected^34–36^. All these mutations can have a huge impact on bacterial fitness, thus affecting the large-scale fermentation of the strains and the OAg production level, whose biosynthesis is closely connected with that of lipid A, thus resulting in GMMA with very low OAg amount with respect to proteins amount^5,37^.

In the present study, we generated a *S. flexneri* scaffold strain suitable for GMMA production and displaying the serotype Y OAg backbone. The scaffold strain was easily converted into 12 different *S. flexneri* serotypes following introduction of the specific OAg-modifying enzymes and their combinations (Fig. 1). Indeed, different genes residing outside the OAg cluster are involved in the chemical modification of the OAg sugar repeats, giving rise to the serological heterogeneity of *S. flexneri* (Supplementary Table 1). To date, 9 OAg-modifying enzymes able to add α-glucose or O-acetyl groups to different sugars in the OAg RU have been described^27^. Six temperate bacteriophages (SfI, SfII, SfIV, SfV, SfX and SfVII) are responsible for acquisition by lysogeny of glucosyltransferase (*gtr*) operons mediating the glucosylation of the OAg backbone. Three bacteriophages (Sf6, SfII and Sf101) are responsible for the acquisition of O-acetyltransferase (*oac*) genes mediating the O-acetylation of the OAg backbone. Moreover, plasmid-borne *opt* genes encoding for phosphoethanolamine (PEtN) transferases were also described^27^. We focused on the introduction of 6 enzymes known to confer the major immuno-determinants (O-factors) recognized by the commercially available Denka Seiken monovalent antisera kit. Indeed, *oacB* and *oacD* are responsible only for minor OAg modifications which differentiate variants of the same serotype and subtype of *S. flexneri*, while *gtrVII* function is restricted to *gtrI*-modified RU. GMMA from selected serotypes were tested in mice and proved to induce high levels of bactericidal antibodies, similarly to GMMA obtained from the corresponding natural serotypes (Fig. 2b).

The findings presented here can impact significantly the overall process to obtain multicomponent GMMA-based vaccines. First, the time needed to obtain GMMA-producing strains representative of the various *S. flexneri* serotypes would be tremendously shortened. Indeed, it could take up to a year to obtain a GMP cell bank of a GMMA-producing strain starting by introducing all mutations needed into a wild type strain. Using the approach described, a well characterized *S. flexneri* GMMA-producing scaffold strain could be ready for serotype conversion and GMP cell bank manufacturing in about one week (time needed for transformations with the desired plasmids). Indeed, we can assume that the growth requirements as well as the fermentation conditions would not change dramatically following serotype conversion, thus facilitating GMP manufacturing. Moreover, we could expect that GMMA Drug Substances (DS) obtained from the scaffold strain converted into different serotypes would be characterized by similar quality attributes like OAg size, Lipid A content but most importantly OAg to protein ratio (Table 1). These shared features would largely simplify the DS analytical characterization and the final formulation of the different GMMA components on aluminum hydroxide adjuvant (Alhydrogel). Finally, considering that the same scaffold strain would be used for ad hoc generation of serotype-specific cell banks, we could envisage a simplified regulatory approval pathway, similarly to that of seasonal influenza vaccines. Nevertheless, beside the proof-of-concept described here, additional considerations should be done before moving to the GMP environment. First of all, the scaffold strain should be further mutated to reduce possible reactogenicity due to the presence of Lipid A while ensuring that the desired OAg to protein ratio in the final GMMA is maintained. Secondly, use of antibiotics for selection and maintainance of the complementation plasmids should be avoided in large scale fermentations. Different strategies to stabilize the plasmids might be used such as the previously described use of the *nadAB* genes to remove the nicotinic acid auxotrophy in *S. sonnei*^4^.

The approach presented here also opened the possibility to combine the different OAg-modifying enzymes in naturally non-occurring ways. Indeed, once serotype Y is converted to a different serotype following lysogeny with a bacteriophage, its potential recipient range for a subsequent lysogeny might be quite different compared to the parental strain^11^. This is evidently due to phage immunity following modification of the OAg, which represents the receptor for the phage adsorption on the cell surface. Accordingly, only certain enzyme combinations occur in nature. On the contrary, our strategy allowed to provide the same bacterium with up to 6 different OAg-modifying enzymes conferring multiple O-factors simultaneously and yielded 16 previously undescribed serotypes (Fig. 3).

While additional work, including 2D NMR experiments, would be needed to fully elucidate the chemical structures of the OAg obtained with our strategy, most of the ^1^H NMR spectra fell into three different categories:

- Hybrid OAg: if two or three enzymes are provided, they are fully compatible in modifying all the RU in the OAg chains
- Mixed OAg: if two enzymes are provided, one is not able to modify a RU which was previously modified by the other enzyme and vice versa
- Mixed and hybrid OAg: if two enzymes are provided, one is able to modify a RU which was previously modified by the other enzyme but not vice versa.

These results clearly show that beside bacteriophage incompatibility, the specificity of each enzyme with the substrate RU plays an important role in the final OAg structure. This is particularly evident for combinations of enzymes like *gtrI* + *gtrIV* and *gtrII* + *oacA* which compete for modifying the same GlcNAc or Rha^I^ on the RU, respectively (Supplementary Table 1, Supplementary Fig. 1). Of note, considering that the Wzy OAg RU polymerase is conserved across all *S. flexneri* serotypes, it is unlikely to obtain different homopolymeric chains when different RU are produced from the same bacterium, but rather heteropolymeric OAg chains (either “mixed” or “mixed and hybrid”).

Most importantly, both GMMA displaying mixed (1 + 2) or hybrid (1 + 3) OAg were able to combine the immunogenicity of 2 serotypes in one single vaccine component. These results were particularly surprising for GMMA displaying the hybrid 1 + 3 serotype as, despite displaying a naturally non-existing polysaccharide, they were able to induce bactericidal antibodies against both serotype 1a and 3a, similarly to physical mixtures of GMMA displaying the individual serotypes (Fig. 4b).

Our results show that the complexity of a GMMA-based *Shigella* vaccine could be minimized by reducing the vaccine components and therefore the cost of goods. Furthermore, by using a GMMA component which co-displays two serotypes we could halve the costs of the fermentation, DS purification and DS characterization processes. Moreover, the formulation process would be simplified and, in the case of hybrid OAg, the amount of LPS and GMMA proteins would be dramatically reduced in the final Drug Product (DP). Analytical characterization of GMMA displaying different OAg combinations might be very challenging, especially when more than 3 OAg-modifying enzymes are expressed at the same time. This could represent an issue in terms of Chemistry Manufacturing and Controls (CMC). However, for specific OAg combinations such as the mixed 1 + 2 and hybrid 1 + 3 serotypes, a relatively easy analytical panel was used and allowed to verify lot to lot consistency at small scale in the OAg structure and relative ratio of the two displayed serotypes (Supplementary Fig. 2).

Finally, this strategy proved to maximize the cross-reactivity between *S. flexneri* serotypes. In a recently published work, we used the GMMA-technology to immunize animal models and generate antisera against 14 *S. flexneri* subtypes from 8 different serotypes that were tested for binding to and bactericidal activity against a panel of 11 *S. flexneri* bacteria lines^28^. Interestingly, most of the observed cross-reactivity could not be attributed to the shared OAg backbone and no natural GMMA was able to induce bactericidal antibodies against the entire panel of *Shigella* strains. However we predicted that a mixture of 2 GMMA (displaying serotypes 1b and 3a OAg) could generate such a response. In the present study we verified that a GMMA displaying the hybrid serotype 1 + 3, therefore similar to a mixture of 1b and 3a GMMA, elicited bactericidal antibodies able to mediate killing of the most epidemiologically relevant *S. flexneri* serotypes (Fig. 5).

In conclusion, although it is not possible to predict how these results will translate to human immunogenicity, the study presented here provided several improvements to the design and development of a multi-component GMMA-based *Shigella* vaccine. Indeed, while a *S. sonnei* GMMA component would be anyway needed in final vaccine composition, this strategy would allow to reduce the number of *S. flexneri* GMMA components. Also, similar strategies could be applied to other serotypically variable pathogens like invasive non-typhoidal *Salmonella* and *Klebsiella pneumoniae* to develop novel and protective vaccines.

## Methods

### Bacterial strains and growth condition

All *Shigella flexneri* strains were obtained from Public Health England (Supplementary Table 2). All bacterial strains and derivative mutants were grown at 30 °C in liquid Luria-Bertani (LB) medium, supplemented with the appropriate antibiotic(s), in a rotary shaker for 16 h. For GMMA production, overnight cultures were diluted in HTMC medium (15 g/L Glycerol, 30 g/L Yeast extract, 0.5 g/L MgSO_4_, 5 g/L KH_2_PO_4_, 20 g/L K_2_HPO_4_), supplemented with the appropriate antibiotic(s), to an optical density at 600 nm (OD600) of 0.3 and grown at 30 °C in a rotary shaker for 8 h using baffled flasks with a liquid to air volume ratio of 1:5. GMMA yields from 50 mL cultures were around 50 μg/mL (total GMMA protein content/ volume of cell-free supernatant at the end of the growth).

### Generation of *S. flexneri* knock-out and recombinant strains

To generate the GMMA-producing mutants, the kanamycin resistance gene *aph* was used to replace the *tolR* gene. The resistance cassette replacement construct was amplified from the pKD4 vector using forward and reverse primers composed of 50 bp homologous to the flanking regions of the gene to be deleted and approximately 20 bp (Supplementary Table 3) at the 3′ end matching the flanking region of the resistance gene^38^. PCR products were purified and were used to transform recombination-prone *S. flexneri* recipient cells carrying pKD46 plasmid^38^. Following the *tolR* gene deletion, the kanamycin resistance gene was removed through FLP-mediated recombination using the pCP20 plasmid to yield markerless mutant strains^38^. To generate the *S. flexneri* scaffold strain, the kanamycin resistance gene *aph* was then used to replace the locus encompassing the *oacD*, *gtrII* and *oacB* genes. Again, following the *oacD, gtrII and oacB* genes deletion, the kanamycin resistance gene was removed through FLP-mediated recombination. The pCOLA- and pACYC-Duet Expression Systems (Novagen) were used to express five glucosyltransferases (*gtrI*, *gtrII*, *gtrIV*, *gtrV*, *gtrX*) and one O-acetyltransferase (*oacA*), either alone or in combinations. All coding DNA sequences (CDS) together with their promoter region were amplified from different *S. flexneri* genomes by polymerase chain reaction (PCR) using KAPA Hi-Fi polymerase (KAPA Biostystems) and specific primer sets (Supplementary Table 3). All amplified CDS and vectors were digested with the specific restriction enzymes (Supplementary Table 3), ligated using T4 DNA ligase (Roche) and transformed into chemically competent *E. coli* DH5α. The plasmids were selected in the presence of 50 µg/ml kanamycin (pCOLA-Duet) or 20 µg/ml chloramphenicol (pACYC-Duet). All generated plasmids were purified from *E. coli* (Supplementary Table 2) and used to transform electro-competent *S. flexneri* recipient cells (Supplementary Table 2).

### FACS analysis

*S. flexneri* strains were grown overnight at 30 °C in LB supplemented with the appropriate antibiotic. Bacteria were then pelleted and washed with PBS at 8000×*g* for 5 min. Bacteria were then blocked with PBS containing 3% (w/v) bovine serum albumin (BSA) for 15 min and incubated with polyclonal rabbit sera (Denka Seiken) diluted in PBS + 1% (w/v) BSA (1:500) for 1 h. After washes, samples were incubated with Alexa Fluor 488 mouse anti-rabbit IgG (1:500) [Molecular Probes] for 30 min. Finally, bacteria were fixed with 4% (w/v) formaldehyde for 20 min and flow cytometry analysis was performed using FACS Canto II flow cytometer (BD Biosciences).

### GMMA production and characterization

After growth in HTMC medium, bacteria were pelleted by centrifugation at 5000×*g* for 45 min. Cell-free supernatants were recovered and filtered through 0.22 μm Stericup filters (Millipore). After ultracentrifugation of filtered supernatants at 175,000×*g* for 2 h at 4 °C, the resulting pellet containing GMMA was washed with PBS, further ultracentrifuged at 175,000×*g* for 2 h and finally resuspended in PBS. GMMA purity was assessed by HPLC-SEC analysis^39^; total protein content was estimated by micro-BCA using bovine serum albumin (BSA) as a reference following the manufacturer’s instructions (Thermo Scientific).

### OAg purification and characterization

OAg extraction from GMMA was performed by acetic acid hydrolysis^5^. OAg populations were characterized by HPLC-SEC with differential refractive index (dRI) detection to estimate the molecular size distribution. OAg peak relative area was estimated and OAg molecular weight (MP) was calculated using dextrans as standards in the range 12-150 kDa. Sugar content was quantified by HPAEC-PAD, after removal of the low molecular weight core molecules by filtration through Amikon 10k^39^. Permethylation of OAg samples was achieved according to the protocol by Harris et al. ^40^, followed by hydrolysis with 2 M trifluoroacetic acid (TFA) at 125 °C for 1 h and derivatization to alditol acetates^41^. Integration values of the areas of the partially methylated alditol acetates (PMAA) were corrected by the effective carbon response factors^42^. Analytical GLC was performed on a Perkin-Elmer Autosystem XL gas chromatograph equipped with a flame ionization detector and using He as carrier gas. An HP-1 capillary column (Agilent Technologies, 30 m) was used to separate PMAA (temperature program: 1 min at 125 °C, 125–240 °C at 4 °C/min, 2 min at 240 °C); GLC–MS analyses were carried out on an Agilent Technologies 7890A gas chromatograph coupled to an Agilent Technologies 5975 C VL MSD, using the same temperature program reported above. ^1^H-NMR spectroscopy was used to confirm OAg identity and structure. All NMR experiments were performed with a Bruker AEON AVANCE III 600 MHz spectrometer equipped with a high-precision temperature controller using a 5 mm QCI CryoProbe. NMR spectra were recorded at 50.0 ± 0.1 °C. The transmitter was set at the water frequency (4.70 ppm). Proton spectra were acquired using a 90° pulse duration automatically calculated and collected with 32 K data points over a 12 ppm spectral width, accumulating 128 scans. Spectra were processed by applying an exponential function to the FID with a line broadening of 0.80 Hz to increase the signal-to-noise ratio and then Fourier transformed. Data acquisition and processing were performed with TopSpin 3.5 software package (Bruker BioSpin).

### Immunogenicity studies in mice

*S. flexneri* GMMA and their combinations were tested in mice. Animal studies were performed at Toscana Life Science Animal Care Facility under the animal project 479/2017-PR 09/06/2017 approved by the Italian Ministry of Health. Five weeks old female CD1 mice (8 per group) were immunized intraperitoneally with 200 μL of vaccine at day 0 and 28. Doses were normalized so that each group received 0.5 µg of serotype-specific OAg. Single sera collected 2 weeks after second injection were tested for SBA against a panel of *S. flexneri* strains using a high throughput assay based on luminescent readout^43^. Results of the assay were expressed as the IC_50_, the reciprocal serum dilution that resulted in a 50% reduction of luminescence and thus corresponding to 50% growth inhibition of the bacteria present in the assay. GraphPad Prism 7 software was used for curve fitting and IC_50_ determination. Titers below the minimum measurable signal were assigned a value of 50, corresponding to half of the first dilution of sera tested. Statistical analysis was conducted comparing groups with the Mann–Whitney unpaired two-tailed *t* test.

### Reporting summary

Further information on research design is available in the Nature Research Reporting Summary linked to this article.

## Supplementary information

Supplementary information

REPORTING SUMMARY

## Data Availability

The authors declare that data supporting the findings of this study are available within the paper and its supplementary information files.
