# Supplementary material for: Rapid generation of Shigella flexneri GMMA displaying natural or new and cross-reactive O-Antigens
Source: NPJ Vaccines. 2022 Jun 30;7:69. doi: 10.1038/s41541-022-00497-7 (PMC9243986; doi:10.1038/s41541-022-00497-7)

Rapid generation of *Shigella flexneri* GMMA displaying natural or new and cross-reactive O-Antigens

Gianmarco Gasperini[1], Maria Michelina Raso[1,3], Fabiola Schiavo[1], Maria Grazia Aruta[1], Neil Ravenscroft[2], Barbara Bellich[3], Paola Cescutti[3], Francesca Necchi[1], Rino Rappuoli[4], Francesca Micoli[1*]

[1]GSK Vaccines Institute for Global Health (GVGH), Siena, Italy

[2]University of Cape Town, Rondebosch, South Africa

[3]Università di Trieste, Trieste, Italy

[4]GSK Vaccines, Siena, Italy

*Corresponding Author, francesca.x.micoli@gsk.com

Supplementary Information

Supplementary Figure 1. ^{1}H-NMR spectra of OAg isolated from all GMMA generated in this study. ^{1}H-NMR spectroscopy was used to confirm OAg identity and structure in all generated strains.

Supplementary Figure 2. Lot to lot consistency. GMMA displaying mixed (1+2) or hybrid (1+3) OAg were produced by 3 independent growths and fully characterized by using HPAEC-PAD and ^{1}H NMR spectroscopy. Anomeric and O-acetyl regions of NMR spectra are reported.

Supplementary Table 1. List of all the *S. flexneri* OAg-modifying enzymes.

Gene	Resulting OAg modification	Conferred serotype specificity
gtrI	α(1-4) glucosylation of GlcNAc	Type I
gtrII	α(1-4) glucosylation of Rha^{I}	Type II
gtrIV	α(1-6) glucosylation of GlcNAc	Type IV
gtrV	α(1-3) glucosylation of Rha^{II}	Type V
gtrX	α(1-3) glucosylation of Rha^{III}	Group 7(8)
oacA	2-O-acetylation of Rha^{I}	Group 6
oacB	3-O-acetylation of Rha^{III}	Group 9
oacD	6-O-acetylation of GlcNAc	Group 10

Supplementary Table 2. List of all the plasmids and strains used in the study.

Plasmids	Reference
pKD46	Datskenko *et al.*, 2000
pKD4	
pCP20	
pCOLA-Duet	Novagen
pACYC-Duet	Novagen
pCOLA-Duet_*gtrI*	This study
pCOLA-Duet_*gtrII*	This study
pCOLA-Duet_*gtrIV*	This study
pCOLA-Duet_*gtrV*	This study
pCOLA-Duet_*gtrX*	This study
pCOLA-Duet_*oacA*	This study
pACYC-Duet_*gtrI*	This study
pACYC-Duet_*gtrII*	This study
pACYC-Duet_*gtrIV*	This study
pACYC-Duet_*gtrV*	This study
pACYC-Duet_*gtrX*	This study
pACYC-Duet_*oacA*	This study

pCOLA-Duet_*gtrI+gtrII*	This study
pACYC-Duet_*gtrIV+gtrV*	This study
**Strains**	**Reference**
S. flexneri serotype 1a isolate H130920139	PHE[1]
S. flexneri serotype 1b isolate H130920140	PHE[1]
S. flexneri serotype 2a isolate H130920142	PHE[1]
S. flexneri serotype 2b isolate H130920143	PHE[1]
S. flexneri serotype 3a isolate H130920144	PHE[1]
S. flexneri serotype 3b isolate H130920145	PHE[1]
S. flexneri serotype 4a isolate H130920147	PHE[1]
S. flexneri serotype 4b isolate H130920148	PHE[1]
S. flexneri serotype 5a isolate H130920150	PHE[1]
S. flexneri serotype 5b isolate H130920151	PHE[1]
S. flexneri serotype 6 isolate H130920152	PHE[1]
S. flexneri serotype X isolate H130920153	PHE[1]
S. flexneri serotype Y isolate H130920154	PHE[1]
S. flexneri 1a Δ*tolR*::*aph*	This study
S. flexneri 2a Δ*tolR*::*frt*	This study
S. flexneri 3a Δ*tolR*::*frt*	This study
S. flexneri 2a Δ*tolR*::*frt* Δ*oacD-gtrII-oacB*::*frt* (“*S. flexneri* scaffold”)	This study
S. flexneri scaffold Δ*tolR*::*frt* pCOLA-Duet_*gtrI*	This study
S. flexneri scaffold Δ*tolR*::*frt* pCOLA-Duet_*gtrII*	This study
S. flexneri scaffold Δ*tolR*::*frt* pCOLA-Duet_*gtrIV*	This study
S. flexneri scaffold Δ*tolR*::*frt* pCOLA-Duet_*gtrV*	This study
S. flexneri scaffold Δ*tolR*::*frt* pCOLA-Duet_*gtrX*	This study
S. flexneri scaffold Δ*tolR*::*frt* pCOLA-Duet_*oacA*	This study
S. flexneri scaffold Δ*tolR*::*frt* pCOLA-Duet_*gtrI* pACYC-Duet_*oacA*	This study
S. flexneri scaffold Δ*tolR*::*frt* pCOLA-Duet_*gtrI* pACYC-Duet_*gtrX*	This study
S. flexneri scaffold Δ*tolR*::*frt* pCOLA-Duet_*gtrII* pACYC_Duet_*gtrX*	This study
S. flexneri scaffold Δ*tolR*::*frt* pCOLA-Duet_*gtrX* pACYC-Duet_*oacA*	This study
S. flexneri scaffold Δ*tolR*::*frt* pCOLA-Duet_*gtrIV* pACYC-Duet_*oacA*	This study
S. flexneri scaffold Δ*tolR*::*frt* pCOLA-Duet_*gtrX* pACYC-Duet_*gtrV*	This study
S. flexneri scaffold Δ*tolR*::*frt* pCOLA-Duet_*gtrI* pACYC-Duet_*gtrII*	This study
S. flexneri scaffold Δ*tolR*::*frt* pCOLA-Duet_*gtrIV* pACYC-Duet_*gtrI*	This study
S. flexneri scaffold Δ*tolR*::*frt* pCOLA-Duet_*gtrI* pACYC-Duet_*gtrV*	This study
S. flexneri scaffold Δ*tolR*::*frt* pCOLA-Duet_*gtrII* pACYC-Duet_*oacA*	This study
S. flexneri scaffold Δ*tolR*::*frt* pCOLA-Duet_*gtrIV* pACYC-Duet_*gtrII*	This study
S. flexneri scaffold Δ*tolR*::*frt* pCOLA-Duet_*gtrII* pACYC-Duet_*gtrV*	This study
S. flexneri scaffold Δ*tolR*::*frt* pCOLA-Duet_*gtrV* pACYC-Duet_*oacA*	This study
S. flexneri scaffold Δ*tolR*::*frt* pCOLA-Duet_*gtrIV* pACYC-Duet_*gtrV*	This study
S. flexneri scaffold Δ*tolR*::*frt* pCOLA-Duet_*gtrIV* pACYC-Duet_*gtrX*	This study
S. flexneri 3a Δ*tolR*::*frt* pCOLA-Duet_*gtrI*	This study
S. flexneri 3a Δ*tolR*::*frt* pCOLA-Duet_*gtrII*	This study

S. flexneri 3a Δ*tolR*::*frt* pCOLA-Duet_*gtrIV*	This study
S. flexneri 3a Δ*tolR*::*frt* pCOLA-Duet_*gtrV*	This study
S. flexneri 3a Δ*tolR*::*frt* pCOLA-Duet_*gtrI+gtrII*	This study
S. flexneri 3a Δ*tolR*::*frt* pACYC-Duet_*gtrIV+gtrV*	This study
S. flexneri 3a Δ*tolR*::*frt* pCOLA-Duet_*gtrI+gtrII* pACYC-Duet_*gtrIV+gtrV*	This study

Supplementary Table 3. List of all the primers used in the study.

Primer name	5'-3' Sequence	Restriction enzyme	DNA template
tolR KO_F	accgccaggcgtttaccgttagcgagagcaacaaggggtaagccatggcc GTGTAGGCTGGAGCTGCTTC	-	pKD4
tolR KO_R	acccgctctctttcaagcaagggaaacgcagatgtttagataggctgcgt CATATGAATATCCTCCTTAG	-	
oacD-gtrII-oacB KO_F	ttttatctgatacatatacacaaatacaggtatatatgattgcgcagata GTCTTGAGCGATTGTGTAGG	-	pKD4
oacD-gtrII-oacB KO_R	tggtttgttttgttaatttgatgaatggcgtctctattaatttaaaagtt TCCTCCTTAGTTCCTATTCC	-	
gtrI_F	cgcggatccAAGCCTTGCAAGCCATTGTG	BamHI	*S. flexneri* 1a
gtrI_R	catgccatggCCCGTCGAAAAAGACGGGTT	NcoI	
gtrII_F	ataagaatgcggccgcAAGCCTTGCAAGCCATTGTG	NotI	*S. flexneri* 2a
gtrII_R	cccaagcttGAGCTTGAAAAAGGGAGGCG	HindIII	
gtrIV_F	ataagaatgcggccgcTGTCACAAATACGGCACAACG	NotI	*S. flexneri* 4a
gtrIV_R	cccaagcttAGAGCAAGATTTAACGCTACATAAA	HindIII	
gtrV_F	cggggtaccACACGTCCCACCACATCAAA	KpnI	*S. flexneri* 5a
gtrV_R	ccgctcgagTCGGATGAAGAAAGAGGCCG	XhoI	
gtrX_F	cggggtaccAACCACTATCGGAAAGCGCA	KpnI	*S. flexneri* 3a
gtrX_R	ccgctcgagGGTTAACGCGCATGCTACTG	XhoI	
oacA_F	ataagaatgcggccgcAGAAACAGAAGCCACTGGAGC	NotI	*S. flexneri* 3a
oacA_R	cccaagcttCTGCGTGGAAAAGAACTCCAC	HindIII	

Supplementary Table 4. GLC-MS on purified OAg.

Linkage	RRT[a]	Relative molar ratio[b]				
		GMMA 1a scaffold	GMMA 2a scaffold	GMMA mixed 1+2	GMMA 3a natural	GMMA hybrid 1+3
2-Rha	0.56	2.0	2.0	2.0	1.0	1.0
3-Rha	0.57	1.0	-	0.4	0.9	0.7
t-Glc	0.61	1.5	1.5	1.4	1.5	2.0
3,4-Rha	0.64	-	1.3	0.7	-	-
2,3-Rha	0.65	-	-	-	1.0	0.7
3-GlcNAc	1.10	-	2.0	0.6	1.3	-
3,4-GlcNAc	1.15	1.5	-	0.6	-	0.8

[a] Relative retention time to per-acetylated inositol; [b] Molar ratios are expressed relative to 2-Rha, as present in the respective RU. Because of the different sensitivity to acid of the monosaccharides and different strength of the glycosidic linkages in the RU, Glc and GlcNAc are overestimated in some cases.

Supplementary References

1 Gentle, A., Ashton, P. M., Dallman, T. J. & Jenkins, C. Evaluation of Molecular Methods for Serotyping Shigella flexneri. *J Clin Microbiol* **54**, 1456-1461, doi:10.1128/JCM.03386-15 (2016).

Scaffold strain converted to natural *S. flexneri* serotypes

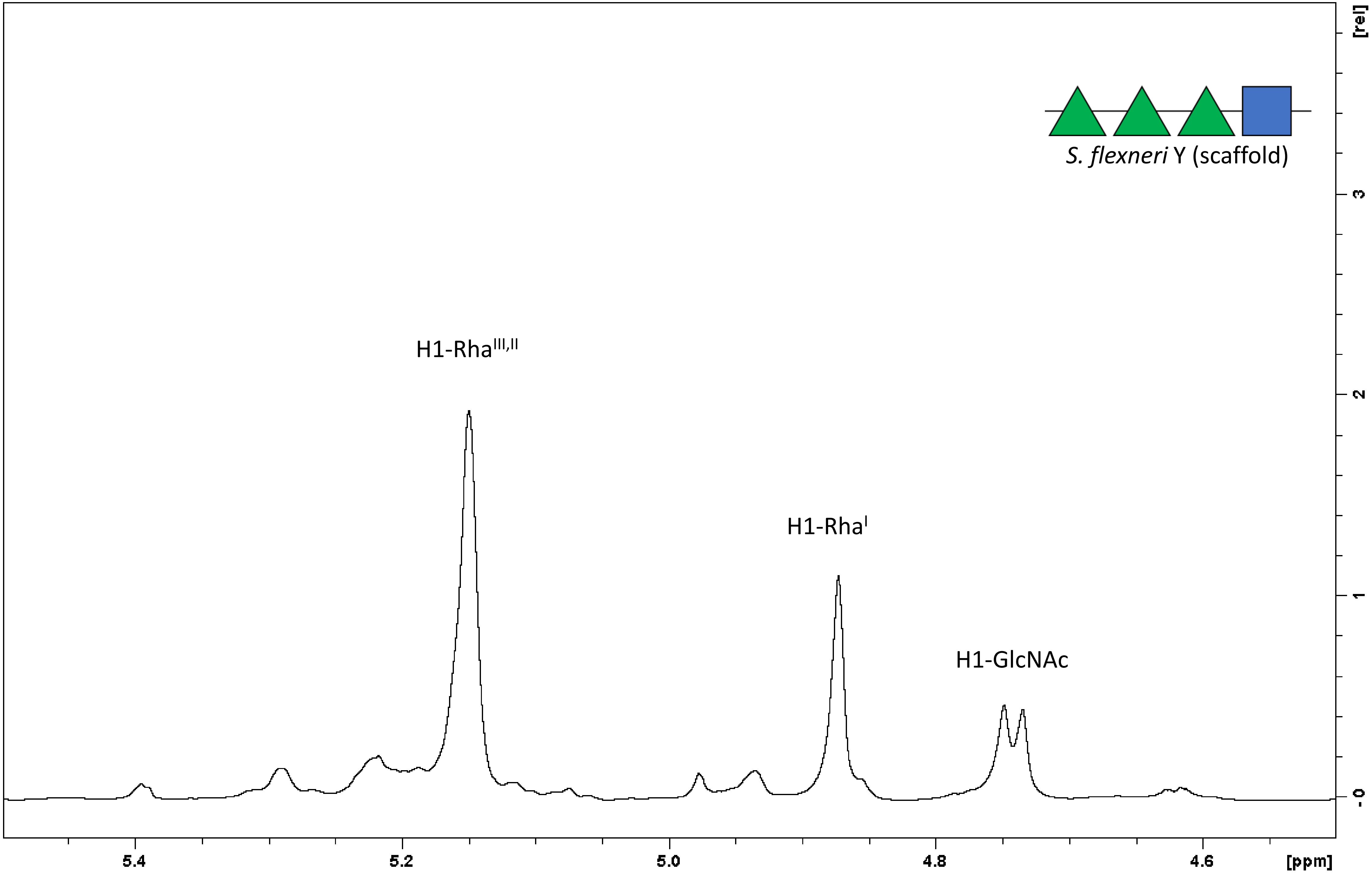
S. flexneri Y (scaffold)
H1-Rha III,II
H1-Rha I
H1-GlcNAc
5.4
5.2
5.0
4.8
4.6
[ppm]
[rel]
3
2
1
0
[1]

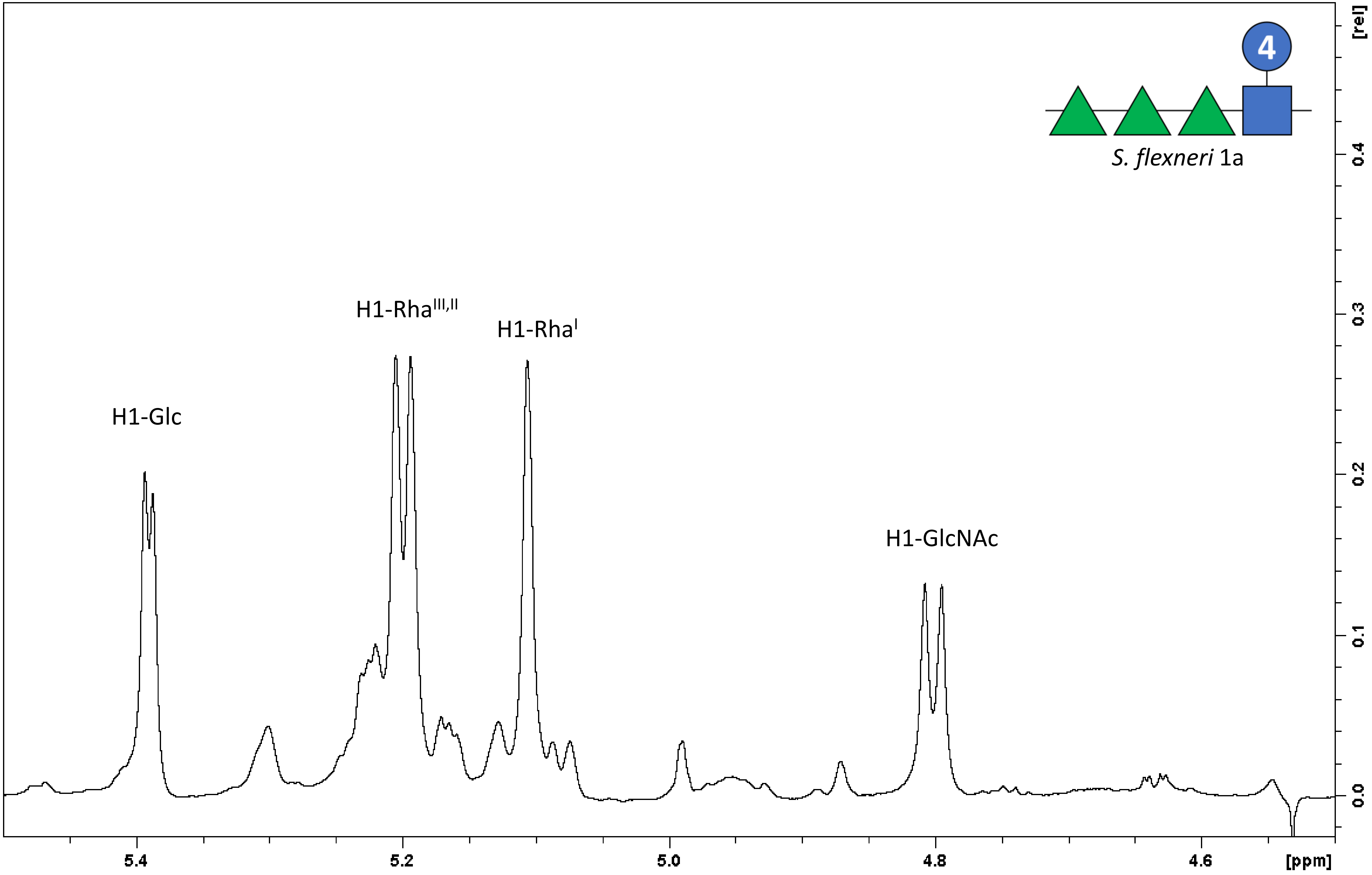
S. flexneri 1a
4
H1-Glc
H1-RhaIII,II
H1-RhaI
H1-GlcNAc
[rel]
0.4
0.3
0.2
0.1
0.0
5.4
5.2
5.0
4.8
4.6
[ppm]

[2]

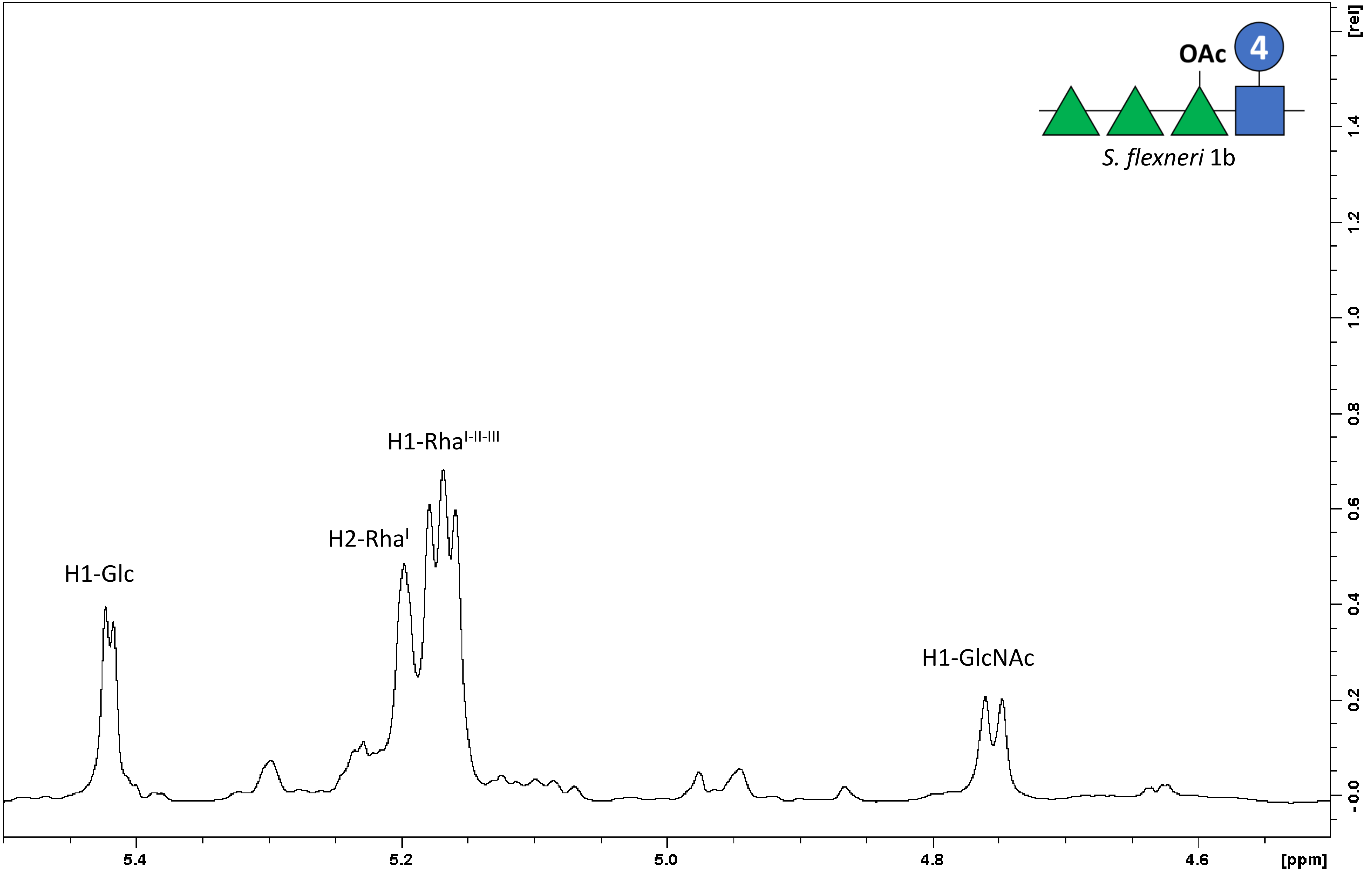
OAc
4
S. flexneri 1b
H1-Glc
H2-Rha[I]
H1-Rha[I-II-III]
H1-GlcNAc
[rel]
[ppm]
5.4
5.2
5.0
4.8
4.6

[2]

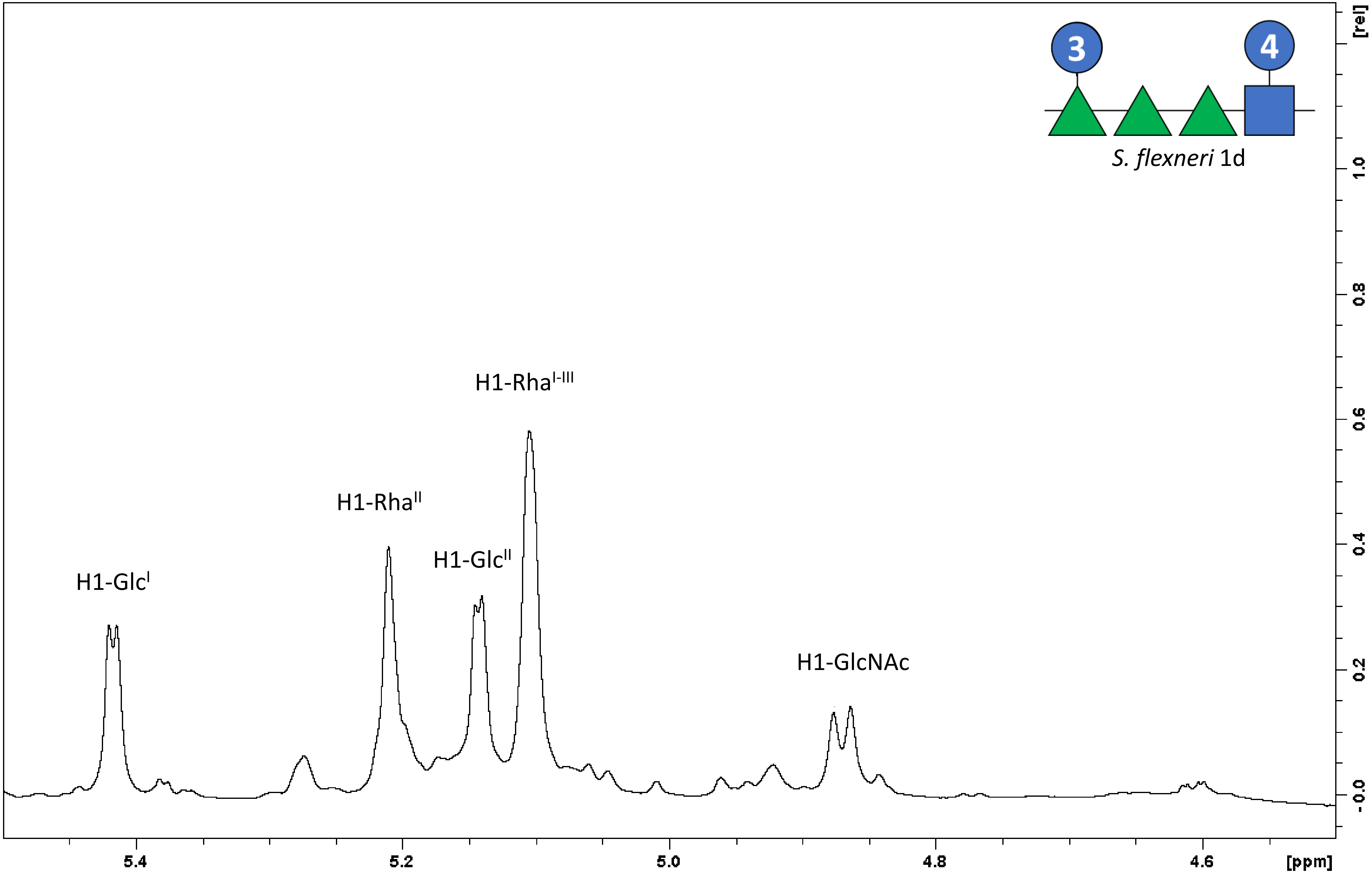
S. flexneri 1d
3
4
H1-Rha I-III
H1-Rha II
H1-Glc II
H1-Glc I
H1-GlcNAc
5.4
5.2
5.0
4.8
4.6
[ppm]
[rel]
1.0
0.8
0.6
0.4
0.2
-0.0

[3]

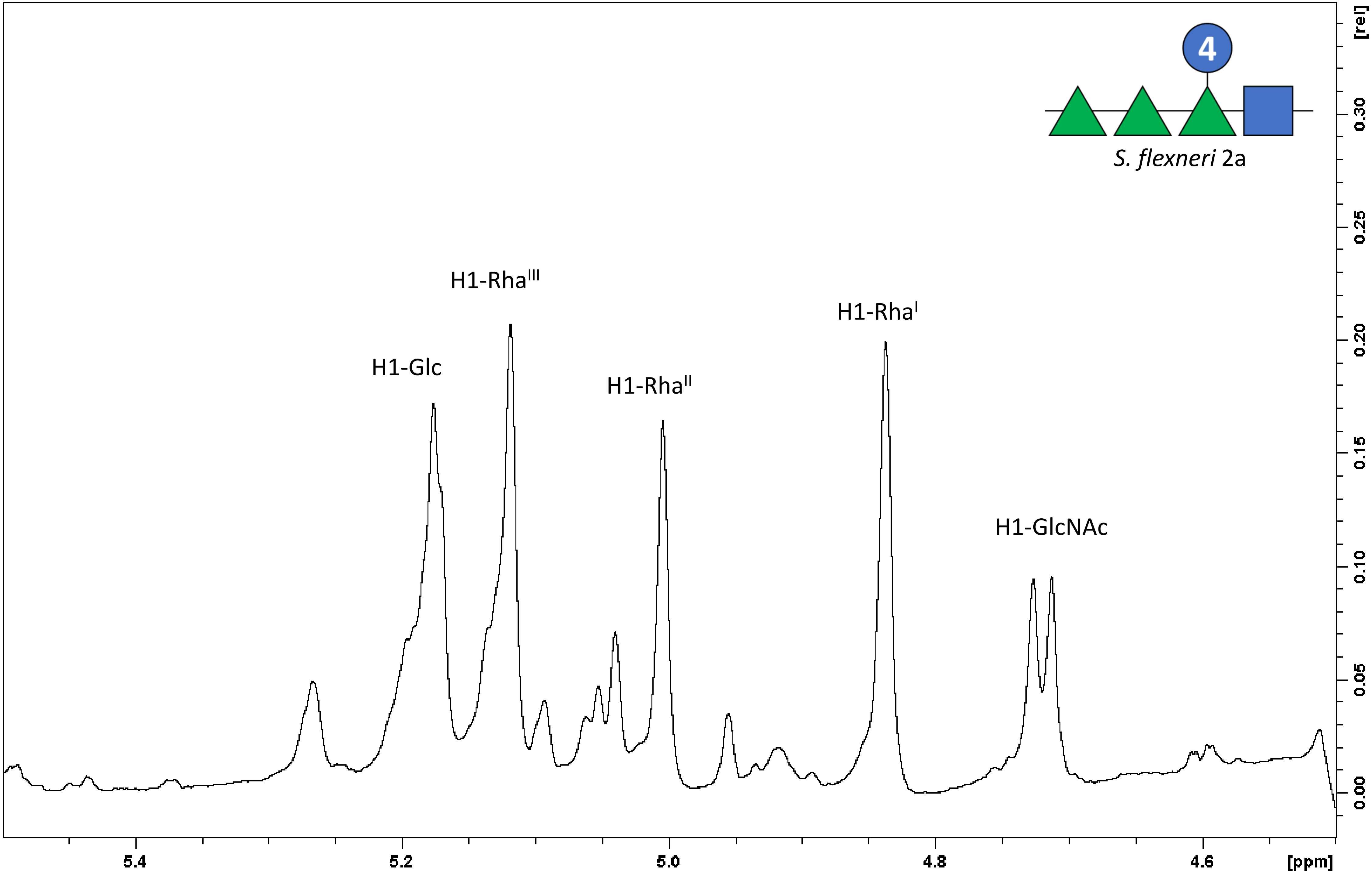
S. flexneri 2a
4
H1-Rha[III]
H1-Glc
H1-Rha[II]
H1-Rha[I]
H1-GlcNAc
[rel]
0.30
0.25
0.20
0.15
0.10
0.05
0.00
5.4
5.2
5.0
4.8
4.6
[ppm]

[2]

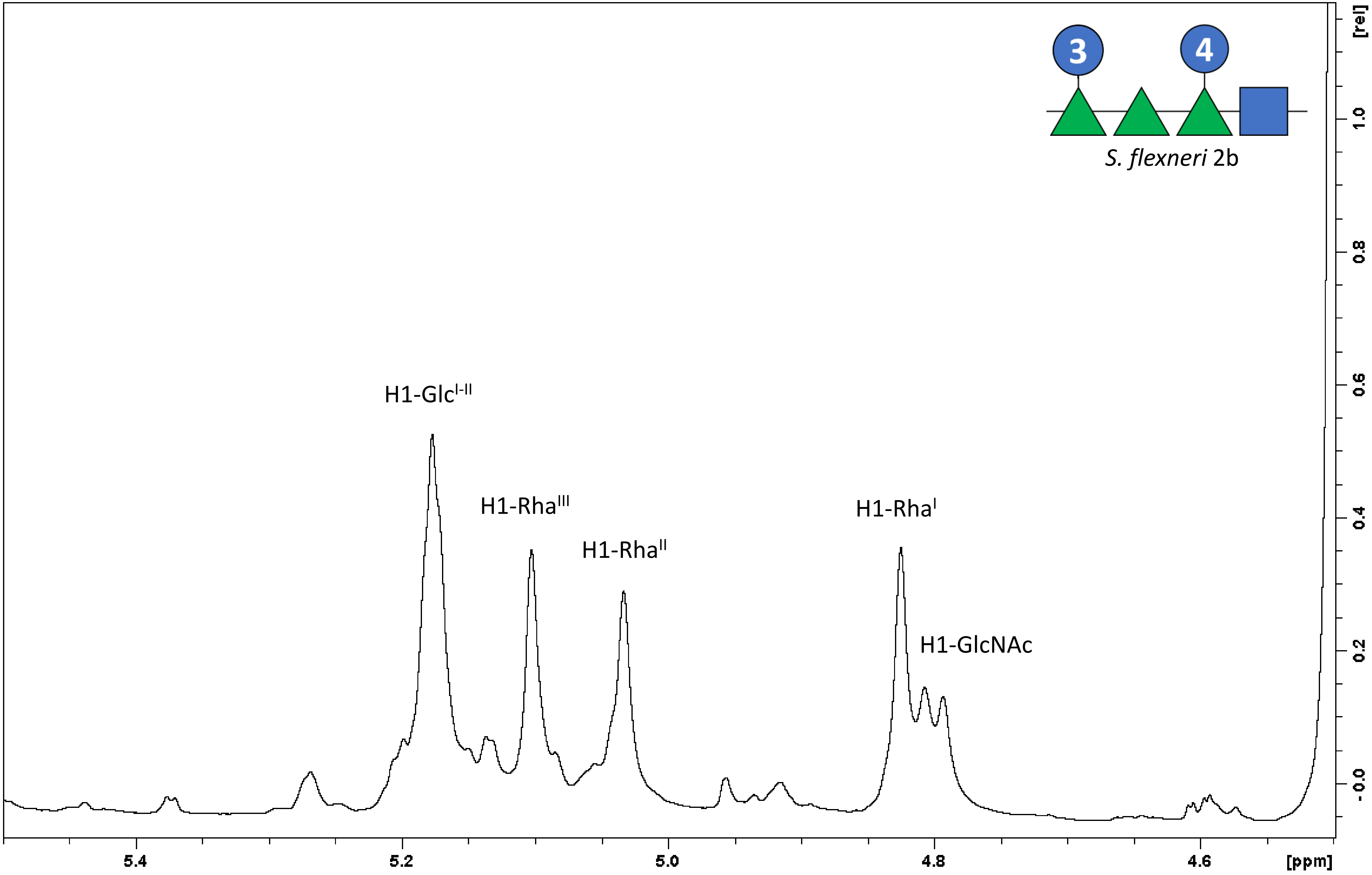

3
4
S. flexneri 2b
H1-Glc I-II
H1-Rha III
H1-Rha II
H1-Rha I
H1-GlcNAc
5.4
5.2
5.0
4.8
4.6
[ppm]
[rel]
1.0
0.8
0.6
0.4
0.2
-0.0

[1]

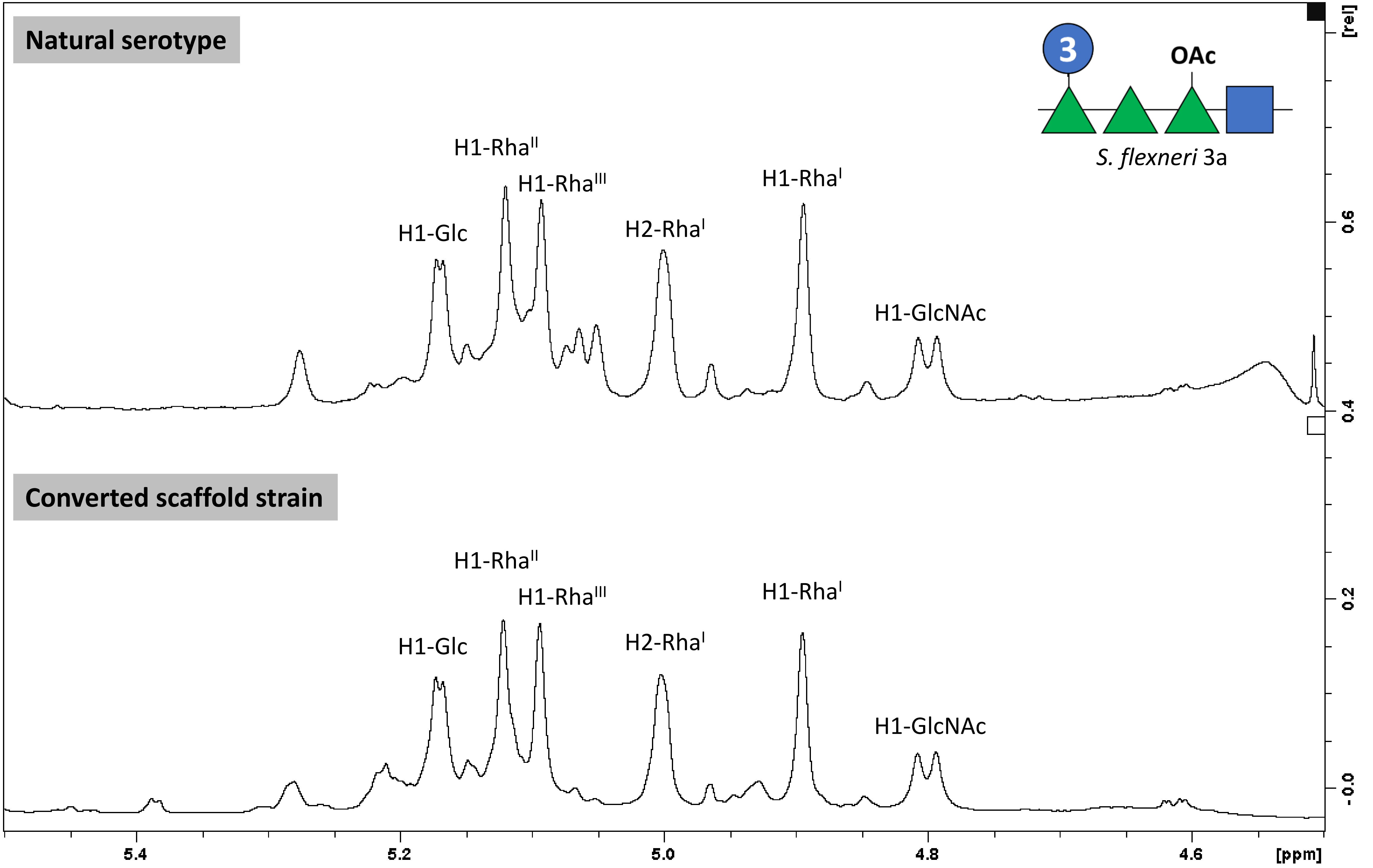
Natural serotype
3
OAc
S. flexneri 3a
H1-Rha^II
H1-Rha^III
H1-Glc
H2-Rha^I
H1-Rha^I
H1-GlcNAc
Converted scaffold strain
H1-Rha^II
H1-Rha^III
H1-Glc
H2-Rha^I
H1-Rha^I
H1-GlcNAc
[rel]
0.6
0.4
0.2
-0.0
5.4
5.2
5.0
4.8
4.6
[ppm]

[1]

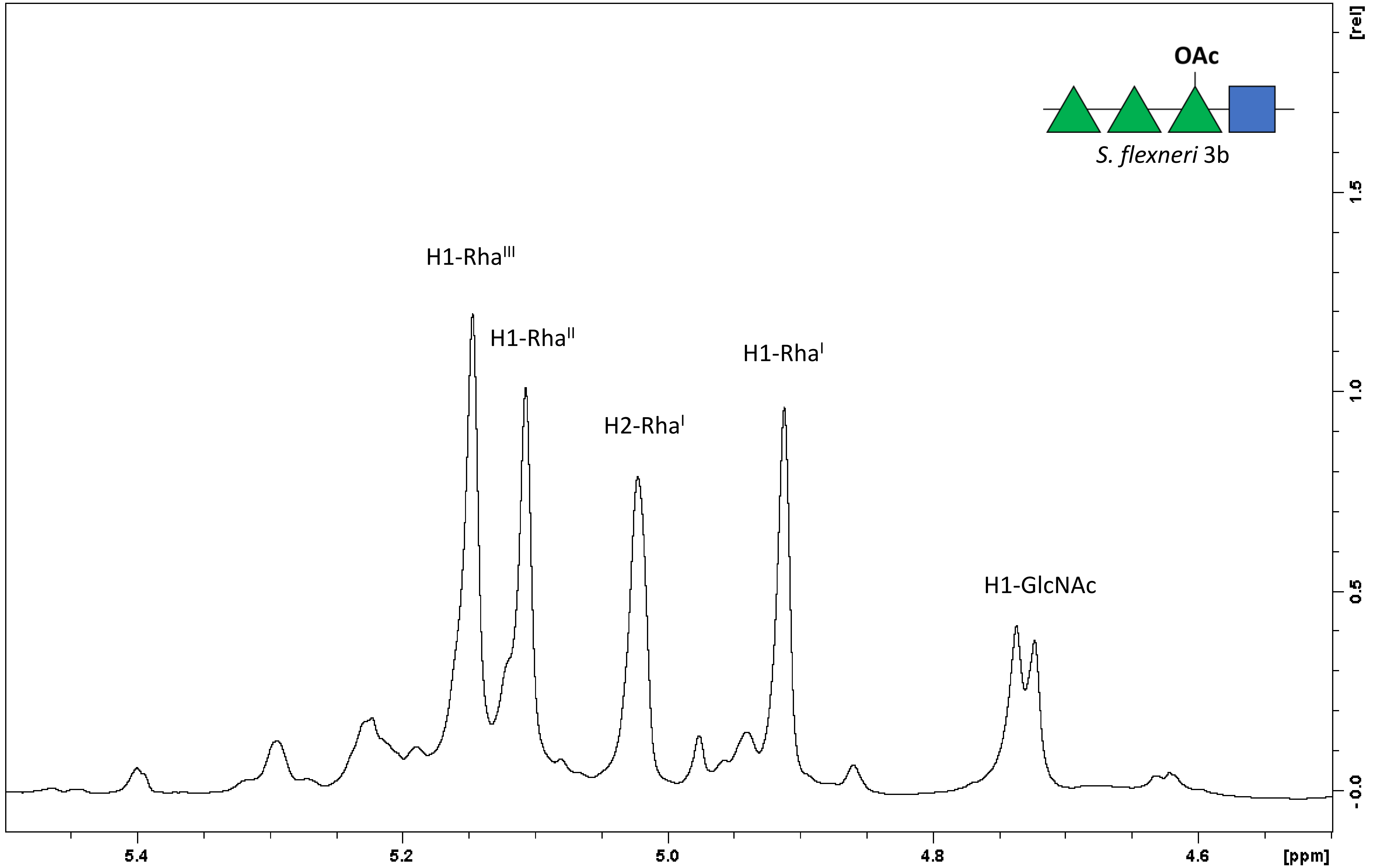
OAc
S. flexneri 3b
H1-Rha^{III}
H1-Rha^{II}
H2-Rha^{I}
H1-Rha^{I}
H1-GlcNAc
[rel]
1.5
1.0
0.5
-0.0
5.4
5.2
5.0
4.8
4.6
[ppm]

[4]

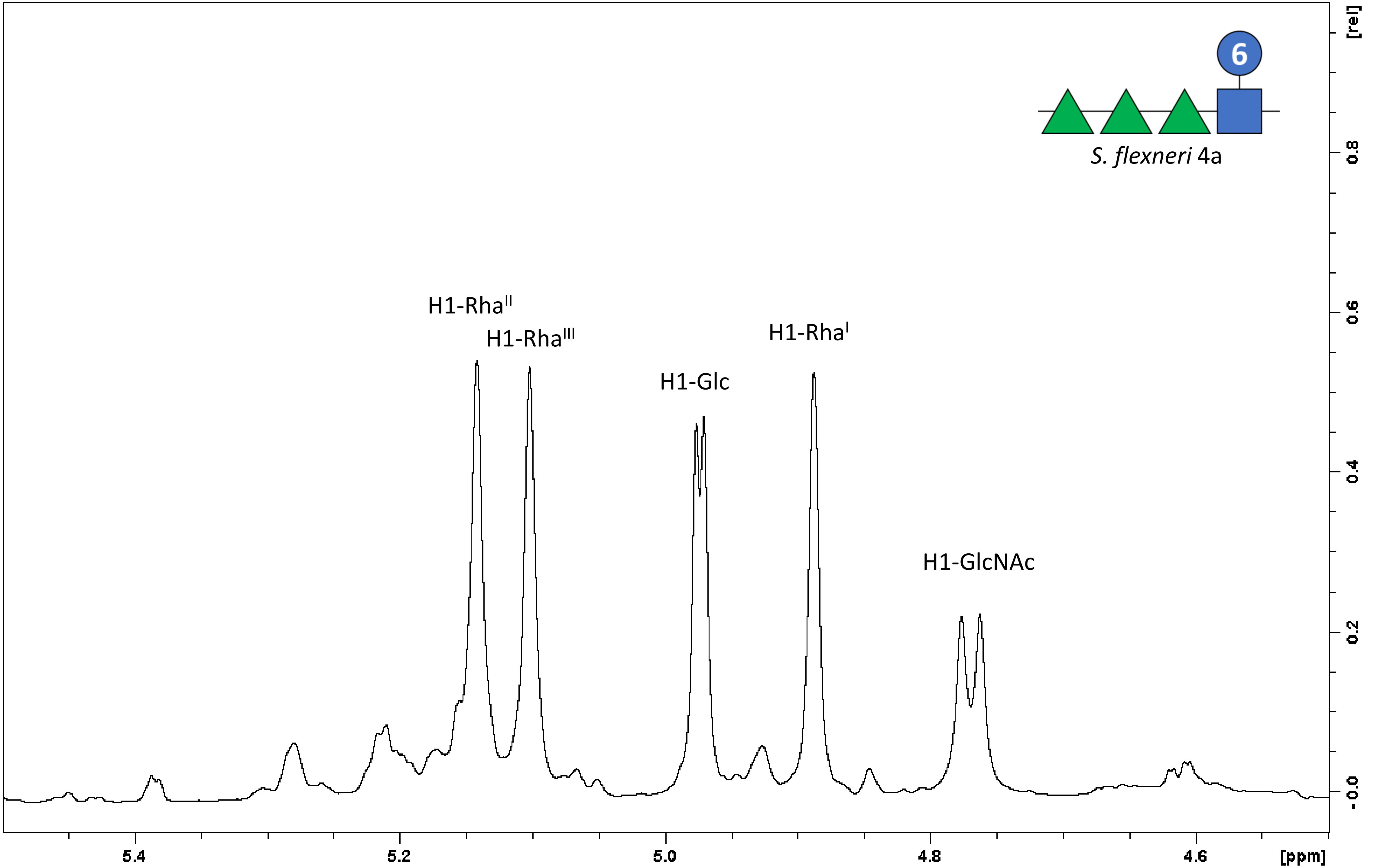

6
S. flexneri 4a
H1-Rha^II
H1-Rha^III
H1-Glc
H1-Rha^I
H1-GlcNAc
[rel]
0.8
0.6
0.4
0.2
-0.0
5.4
5.2
5.0
4.8
4.6
[ppm]

[4]

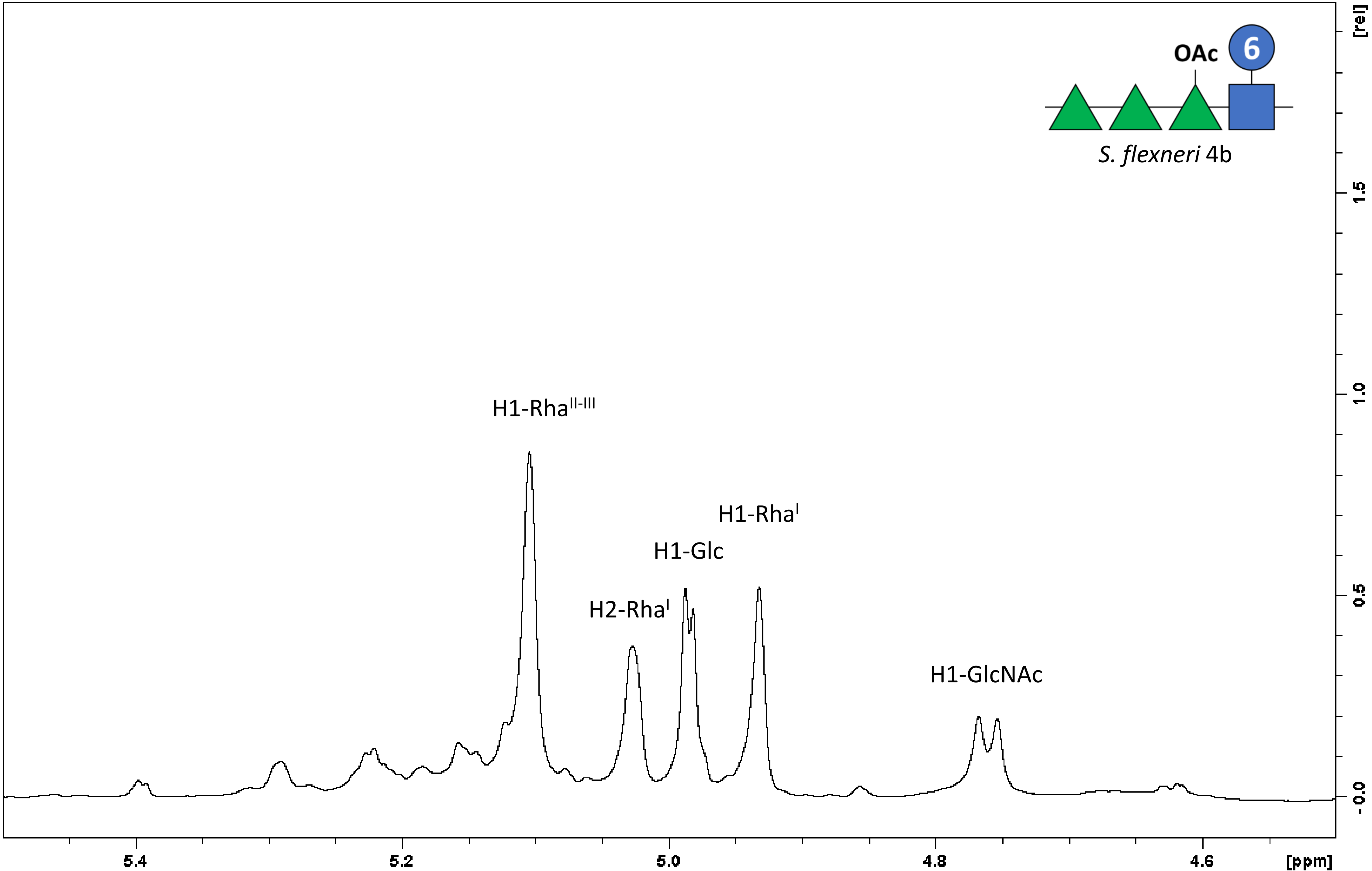
OAc
6
S. flexneri 4b
H1-Rha II-III
H1-Rha I
H1-Glc
H2-Rha I
H1-GlcNAc
[rel]
1.5
1.0
0.5
-0.0
5.4
5.2
5.0
4.8
4.6
[ppm]

[4]

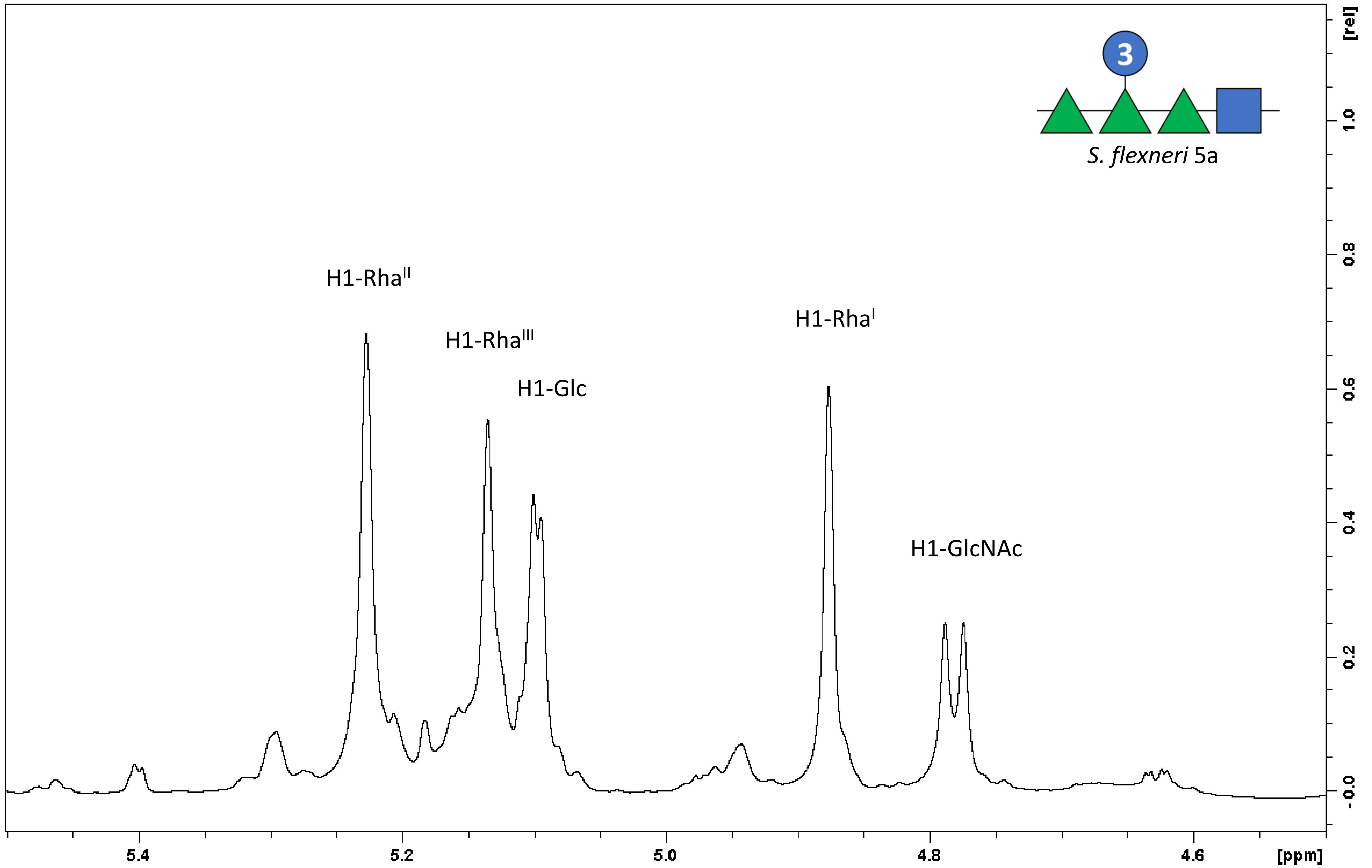
3
S. flexneri 5a
H1-Rha^II
H1-Rha^III
H1-Glc
H1-Rha^I
H1-GlcNAc
[rel]
1.0
0.8
0.6
0.4
0.2
-0.0
5.4
5.2
5.0
4.8
4.6
[ppm]
[4]

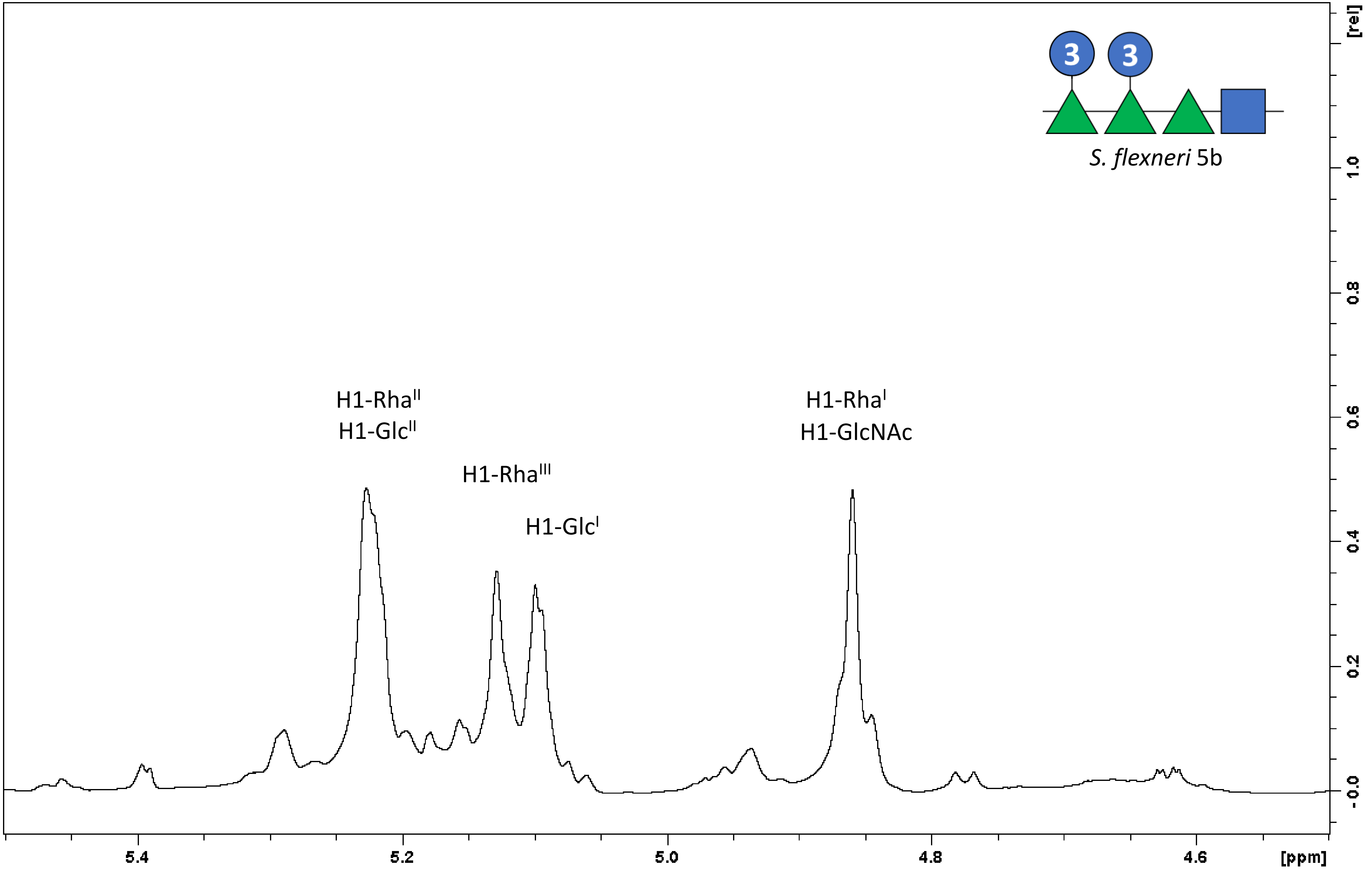
3
3
S. flexneri 5b
H1-Rha^{II}
H1-Glc^{II}
H1-Rha^{III}
H1-Glc^{I}
H1-Rha^{I}
H1-GlcNAc
5.4
5.2
5.0
4.8
4.6
[ppm]
[rel]
1.0
0.8
0.6
0.4
0.2
-0.0

[1]

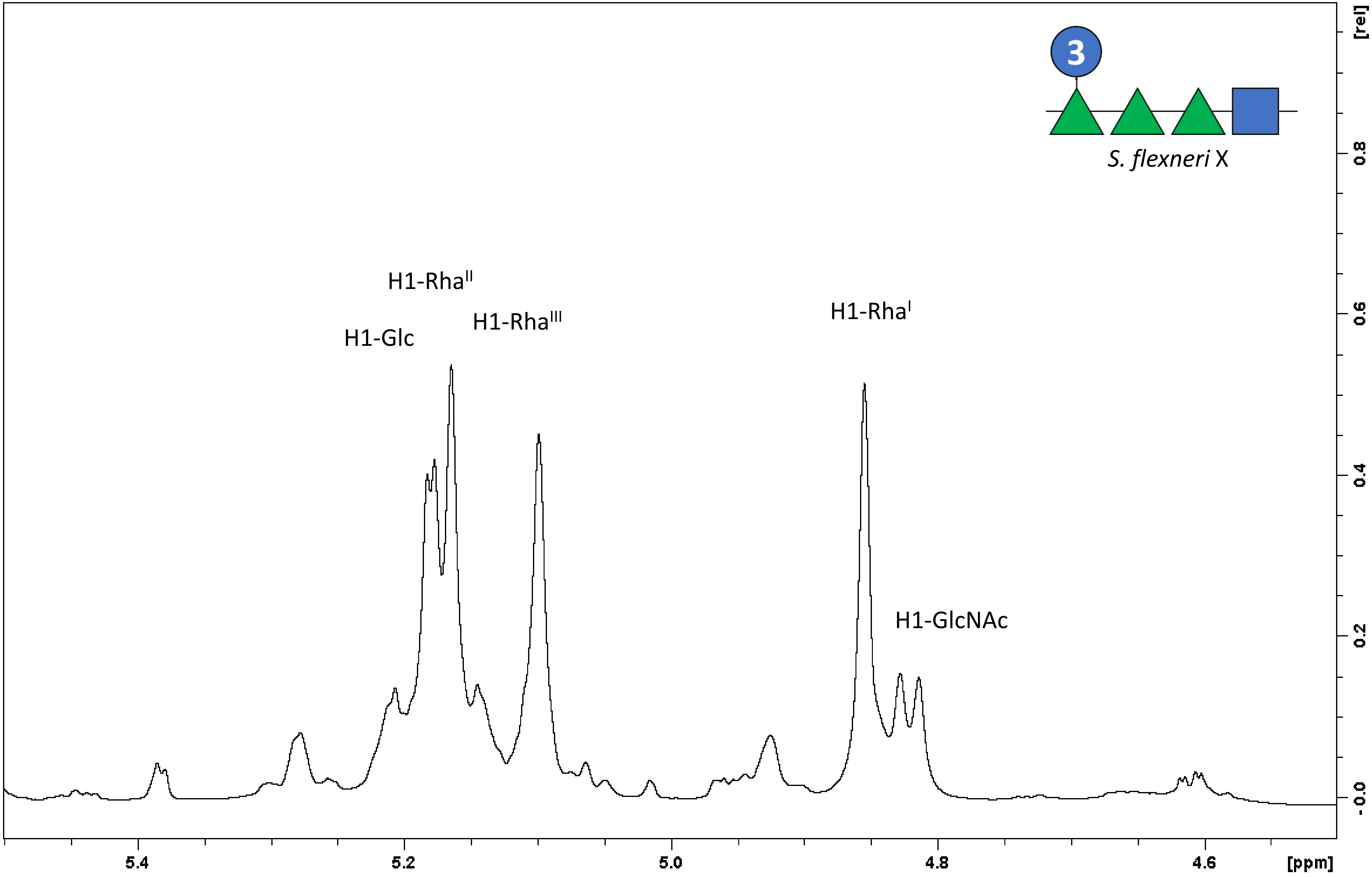
3
S. flexneri X
H1-Rha^II
H1-Glc
H1-Rha^III
H1-Rha^I
H1-GlcNAc
[rel]
0.8
0.6
0.4
0.2
-0.0
5.4
5.2
5.0
4.8
4.6
[ppm]

[4]

Scaffold strain converted to unnatural *S. flexneri* serotypes

Hybrid repeating units

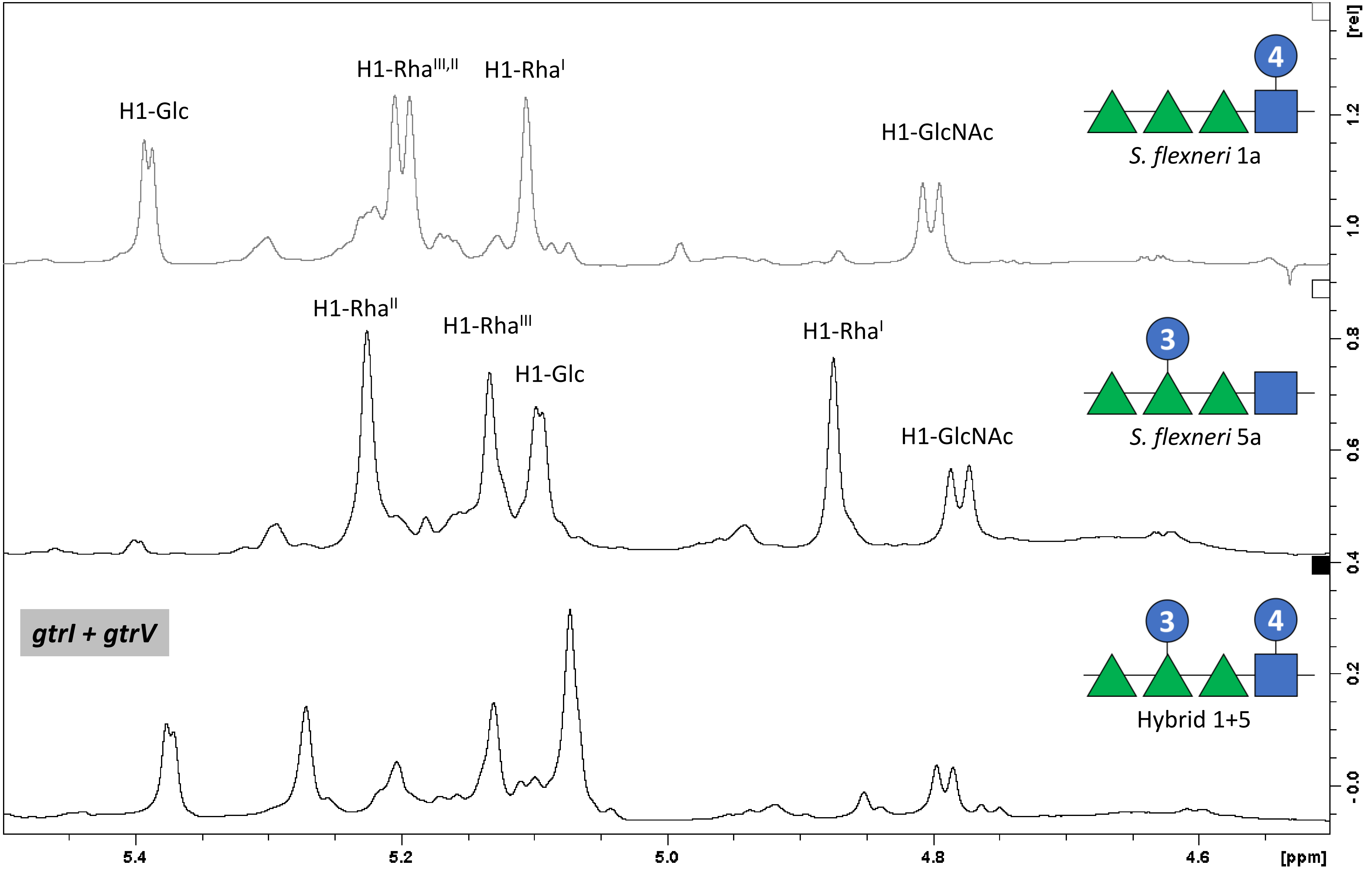
H1-Glc
H1-Rha^{III,II}
H1-Rha^{I}
H1-GlcNAc
S. flexneri 1a
4
H1-Rha^{II}
H1-Rha^{III}
H1-Glc
H1-Rha^{I}
H1-GlcNAc
S. flexneri 5a
3
gtrI + gtrV
Hybrid 1+5
3
4
[rel]
1.2
1.0
0.8
0.6
0.4
0.2
-0.0
5.4
5.2
5.0
4.8
4.6
[ppm]

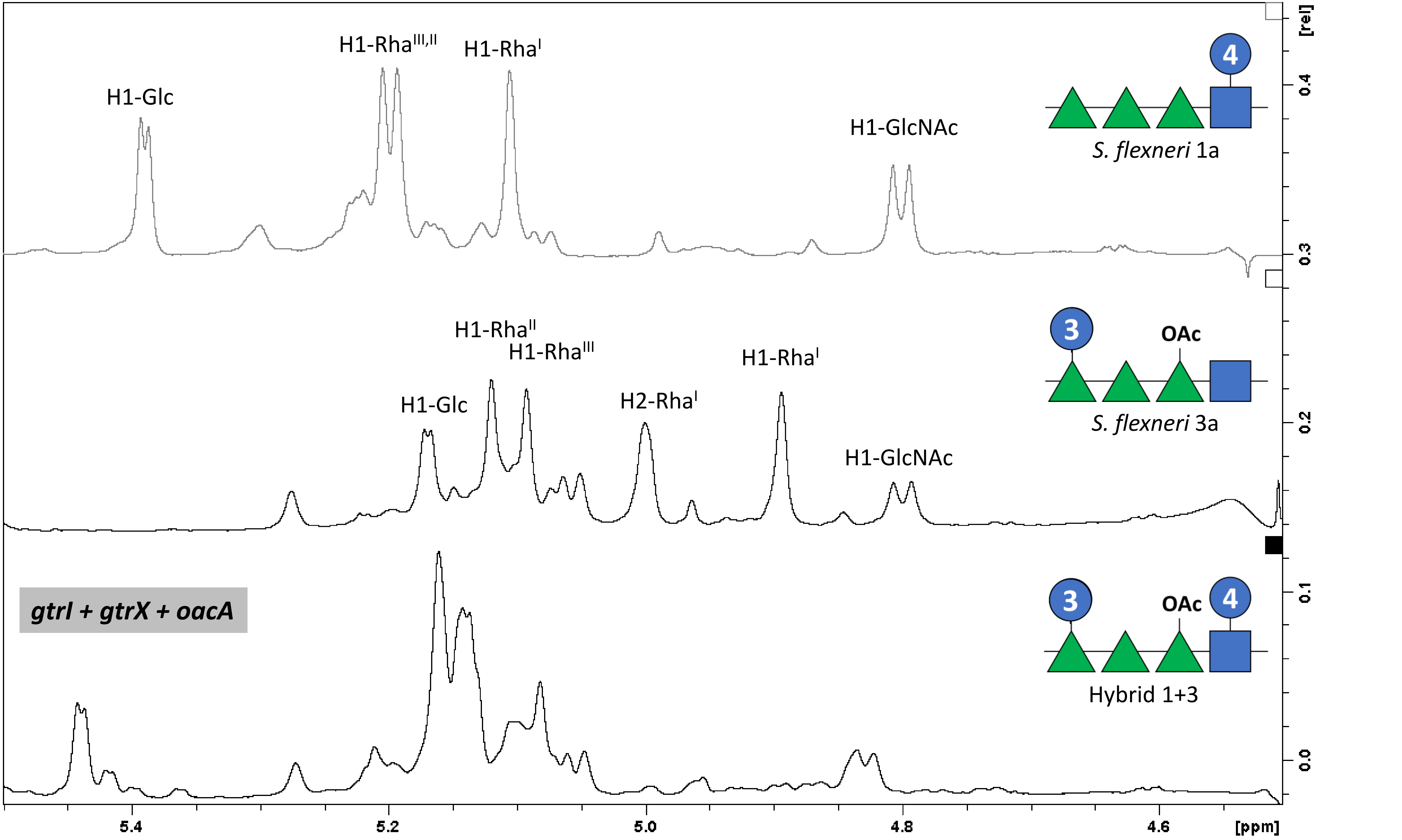
H1-Glc
H1-Rha^{III,II}
H1-Rha^{I}
H1-GlcNAc
4
S. flexneri 1a
H1-Rha^{II}
H1-Rha^{III}
H1-Glc
H2-Rha^{I}
H1-Rha^{I}
H1-GlcNAc
3
OAc
S. flexneri 3a
gtrI + gtrX + oacA
3
OAc
4
Hybrid 1+3
[rel]
0.4
0.3
0.2
0.1
0.0
5.4
5.2
5.0
4.8
4.6
[ppm]

Scaffold strain converted to unnatural *S. flexneri* serotypes

Mixed repeating units

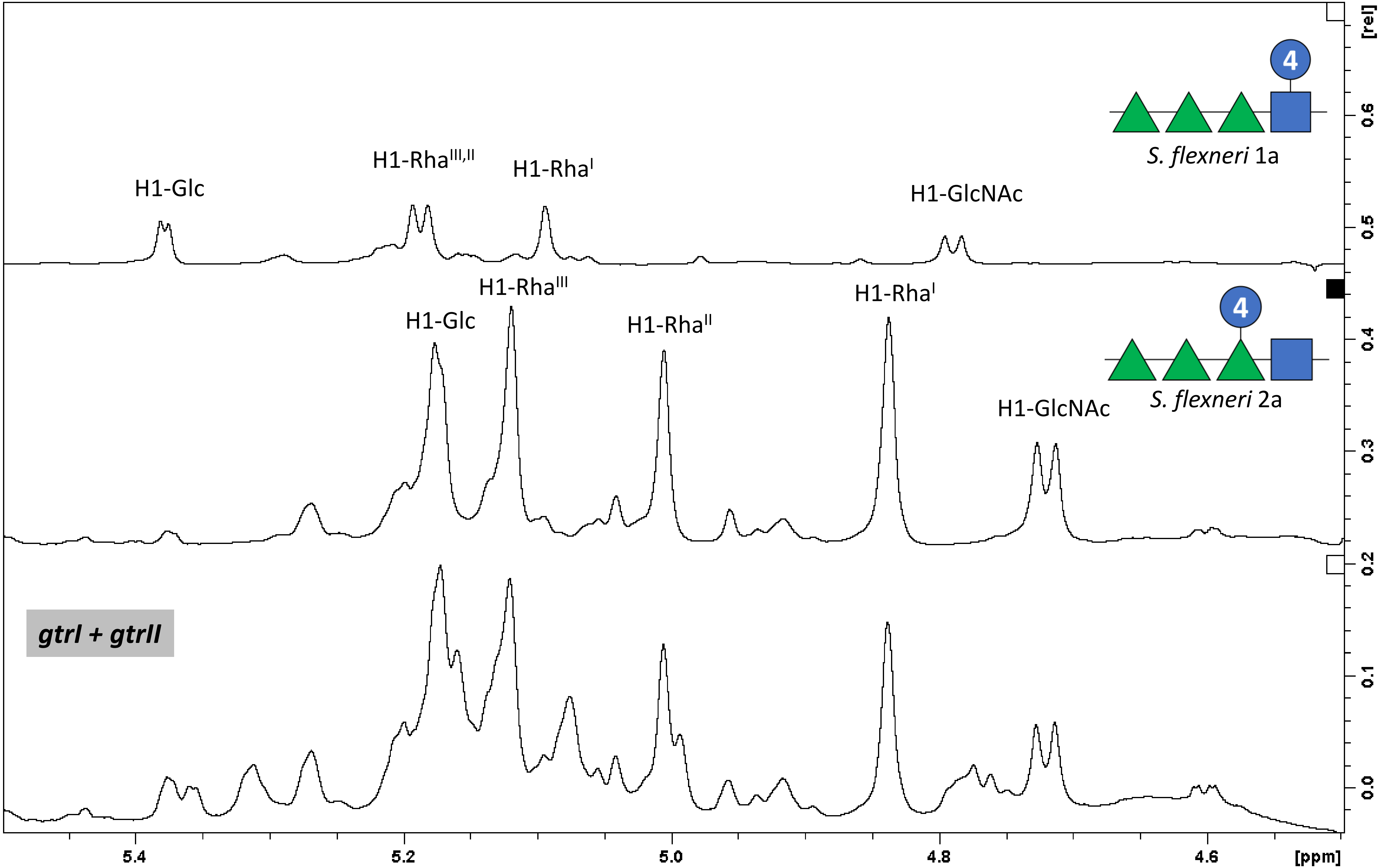

H1-Glc
H1-Rha^{III,II}
H1-Rha^{I}
H1-GlcNAc
4
S. flexneri 1a
H1-Glc
H1-Rha^{III}
H1-Rha^{II}
H1-Rha^{I}
H1-GlcNAc
4
S. flexneri 2a
gtrI + gtrII
5.4
5.2
5.0
4.8
4.6
[ppm]
[rel]
0.6
0.5
0.4
0.3
0.2
0.1
0.0

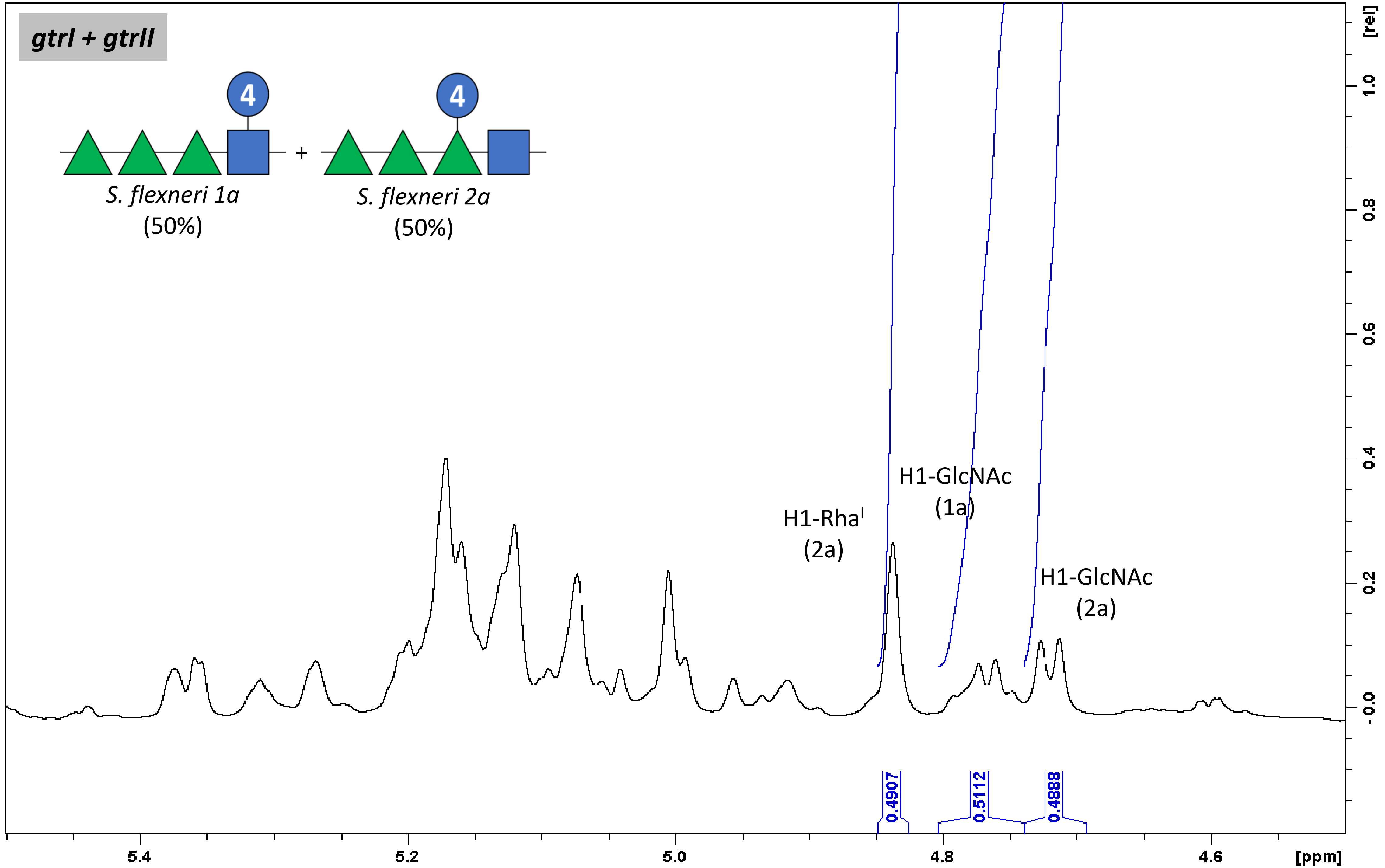
gtrI + gtrII
4
4
+
S. flexneri 1a
(50%)
S. flexneri 2a
(50%)
H1-Rha^{I}
(2a)
H1-GlcNAc
(1a)
H1-GlcNAc
(2a)
0.4907
0.5112
0.4888
5.4
5.2
5.0
4.8
4.6
[ppm]
[rel]
1.0
0.8
0.6
0.4
0.2
-0.0

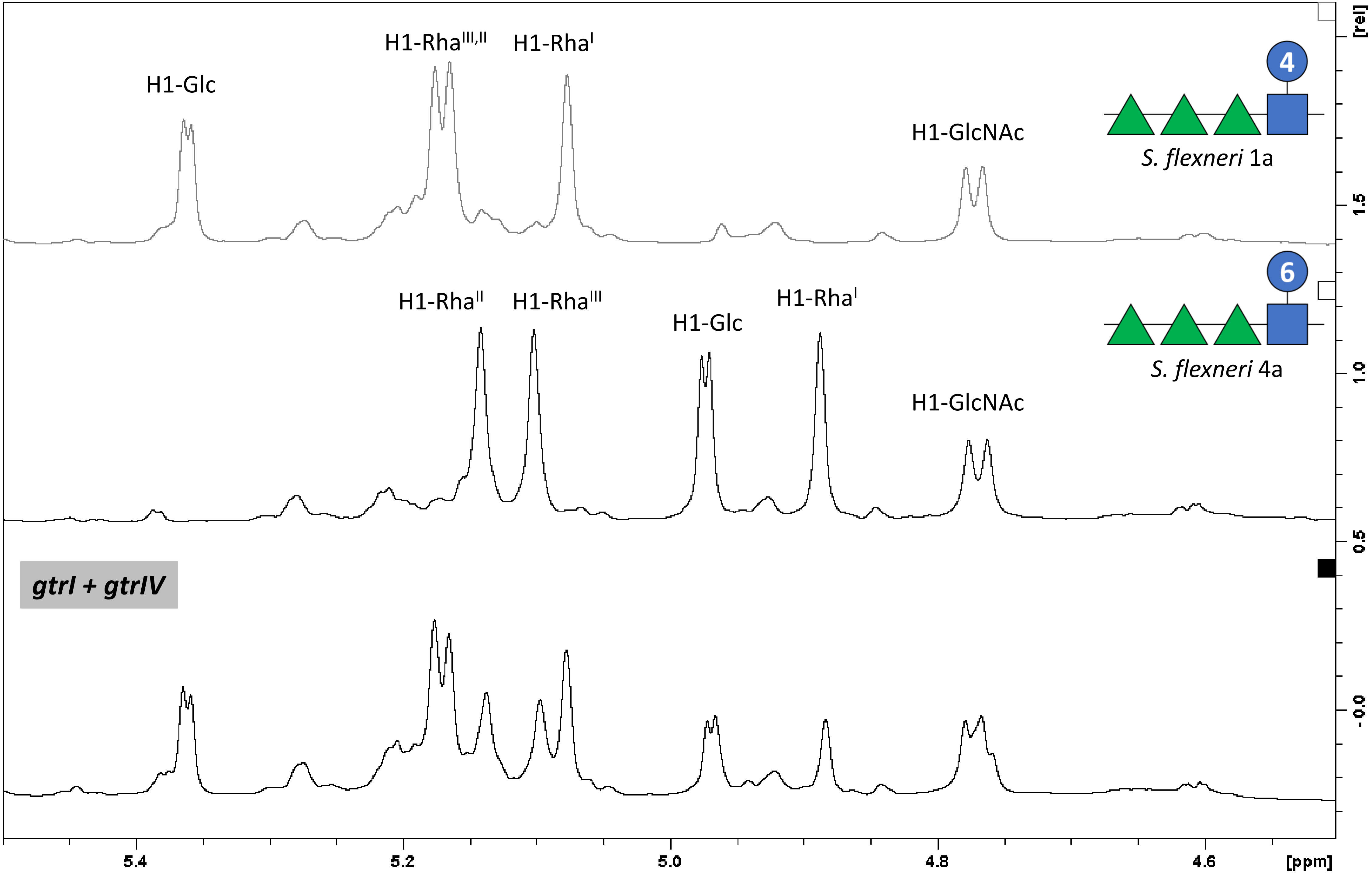

H1-Glc
H1-Rha^III,II
H1-Rha^I
H1-GlcNAc
4
S. flexneri 1a
6
H1-Rha^II
H1-Rha^III
H1-Glc
H1-Rha^I
H1-GlcNAc
S. flexneri 4a
gtrI + gtrIV
5.4
5.2
5.0
4.8
4.6
[ppm]
[rel]
1.5
1.0
0.5
-0.0

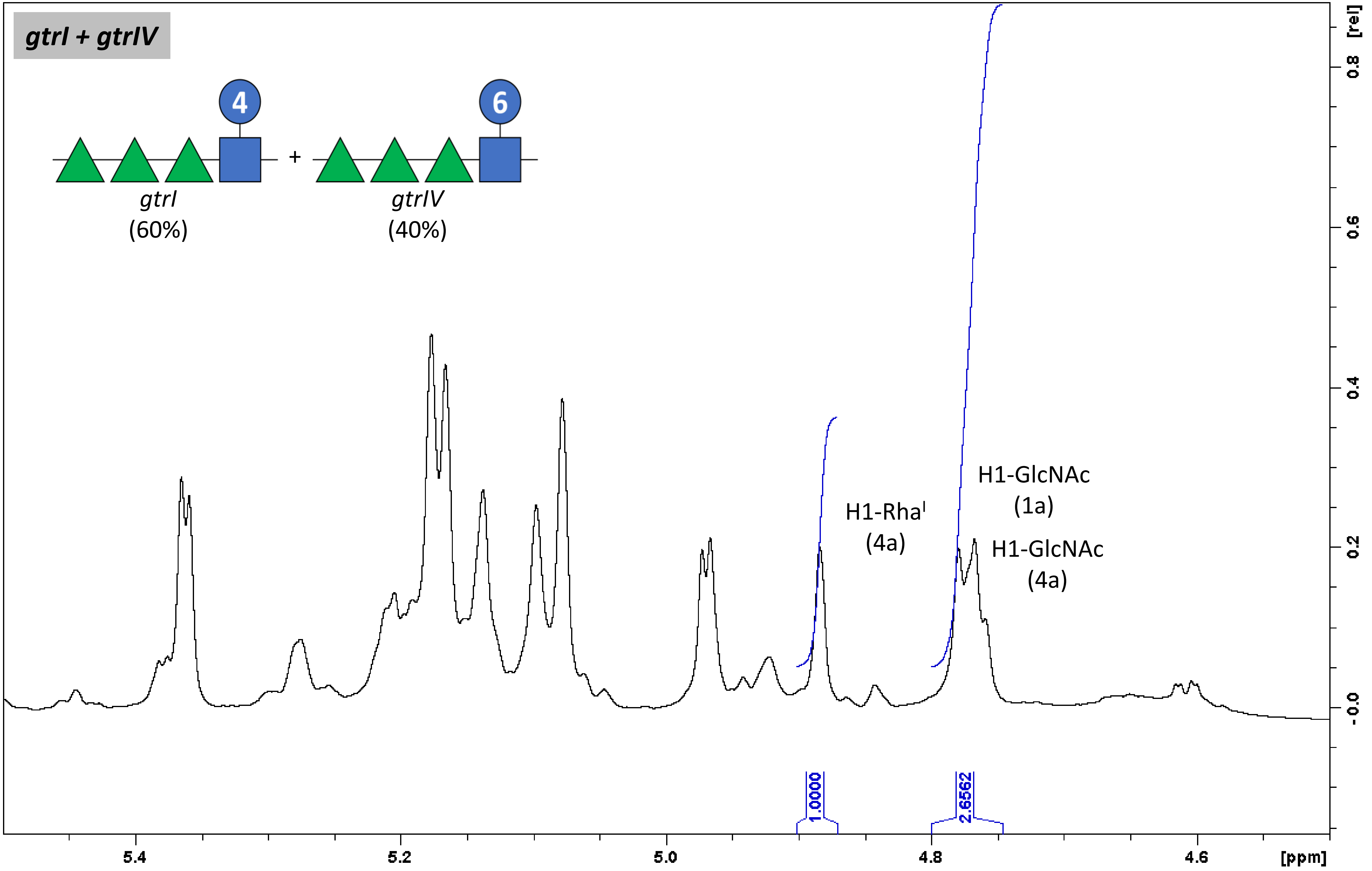
gtrI + gtrIV
4
6
+
gtrI
(60%)
gtrIV
(40%)
H1-GlcNAc
(1a)
H1-Rha^I
(4a)
H1-GlcNAc
(4a)
1.0000
2.6562
5.4
5.2
5.0
4.8
4.6
[ppm]
[rel]
0.8
0.6
0.4
0.2
-0.0

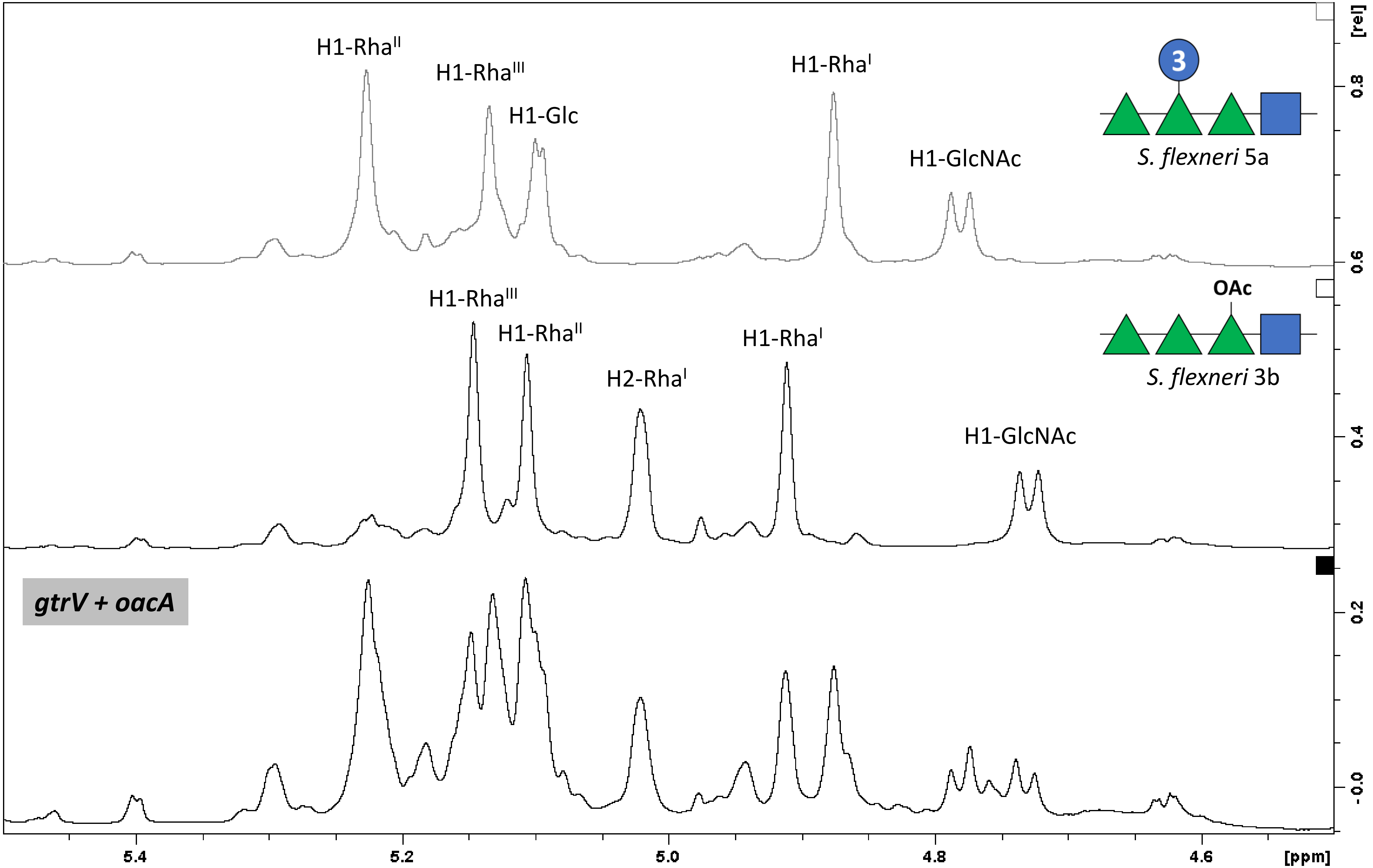

H1-Rha^{II}
H1-Rha^{III}
H1-Glc
H1-Rha^{I}
H1-GlcNAc
3
S. flexneri 5a
H1-Rha^{III}
H1-Rha^{II}
H2-Rha^{I}
H1-Rha^{I}
H1-GlcNAc
OAc
S. flexneri 3b
gtrV + oacA
[rel]
0.8
0.6
0.4
0.2
-0.0
5.4
5.2
5.0
4.8
4.6
[ppm]

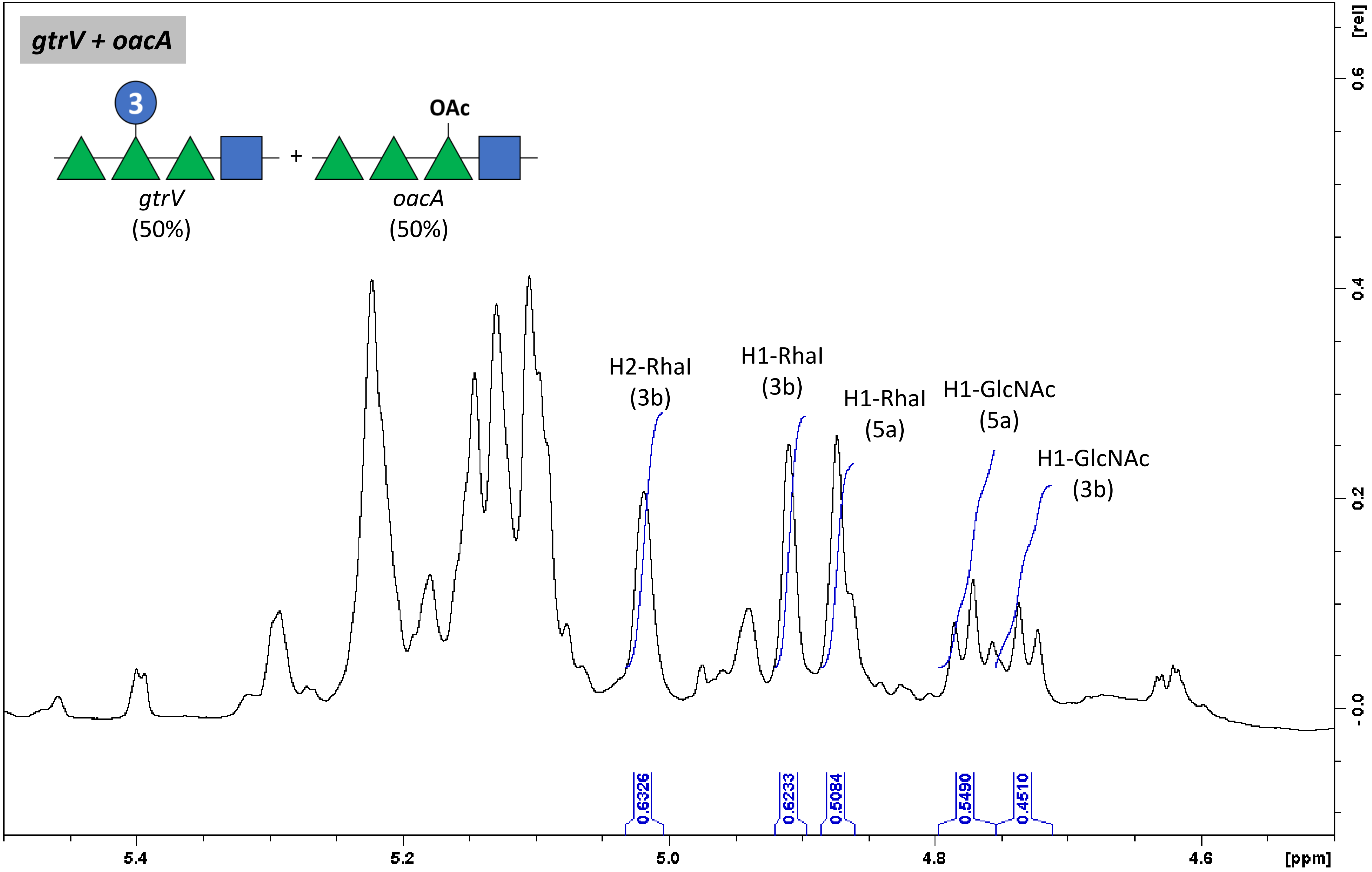

gtrV + oacA
3
OAc
+
gtrV
(50%)
oacA
(50%)
H2-RhaI
(3b)
H1-RhaI
(3b)
H1-RhaI
(5a)
H1-GlcNAc
(5a)
H1-GlcNAc
(3b)
0.6326
0.6233
0.5084
0.5490
0.4510
5.4
5.2
5.0
4.8
4.6
[ppm]
[rel]
0.6
0.4
0.2
-0.0

Scaffold strain converted to unnatural *S. flexneri* serotypes

Mixed and hybrid repeating units

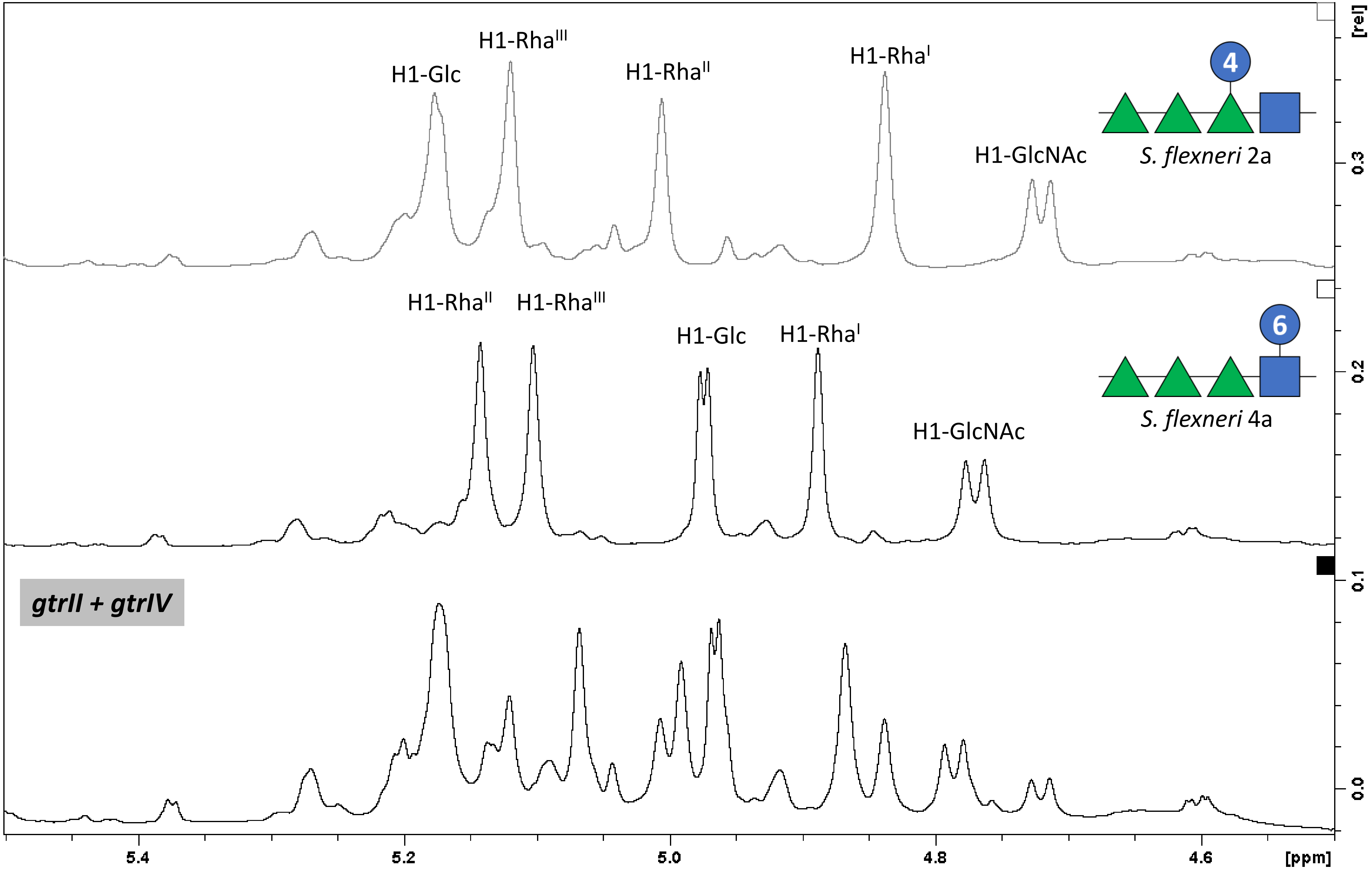
H1-Glc
H1-Rha^III
H1-Rha^II
H1-Rha^I
H1-GlcNAc
4
S. flexneri 2a
H1-Rha^II
H1-Rha^III
H1-Glc
H1-Rha^I
H1-GlcNAc
6
S. flexneri 4a
gtrII + gtrIV
5.4
5.2
5.0
4.8
4.6
[ppm]
[rel]
0.3
0.2
0.1
0.0

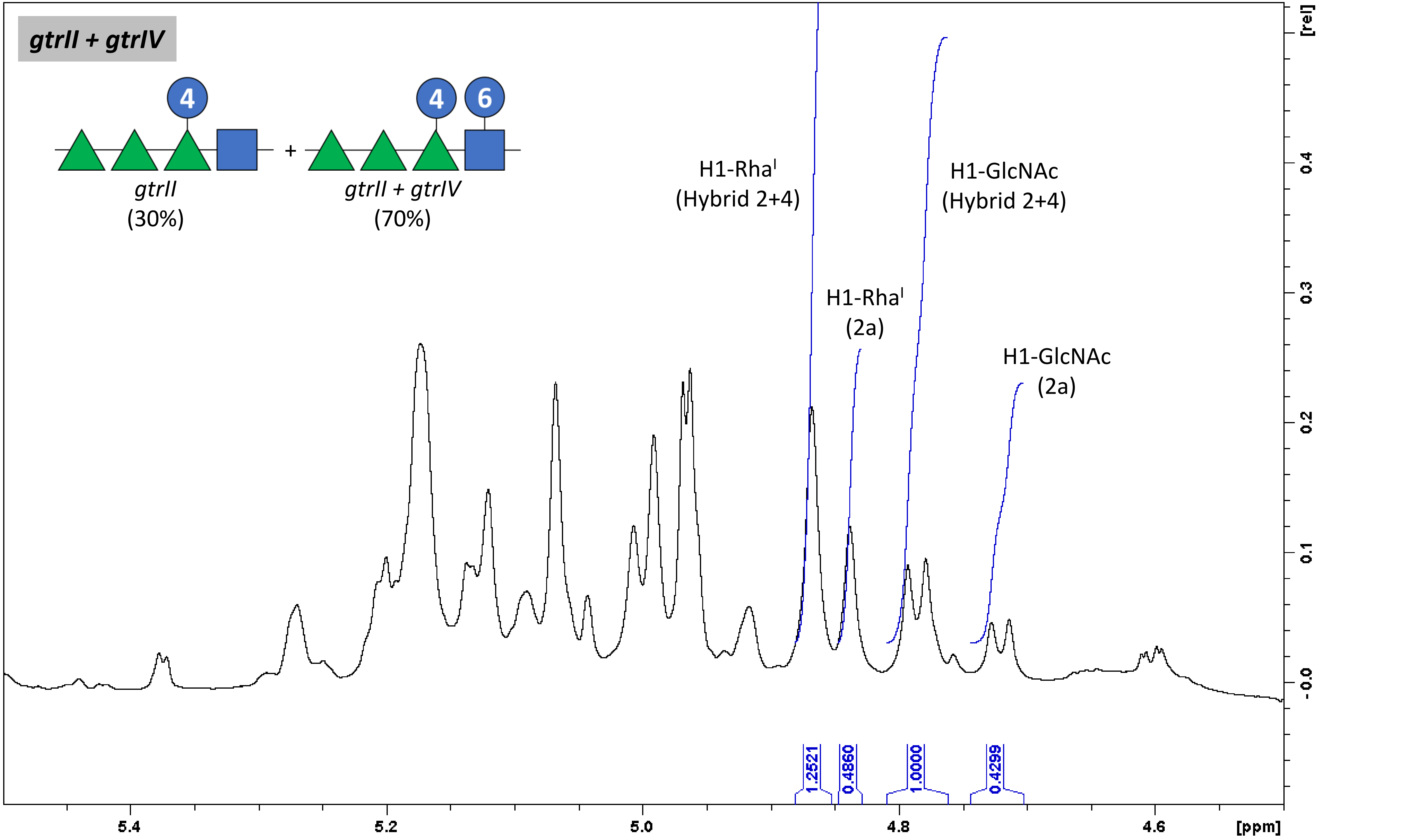
gtrII + gtrIV
4
4
6
+
gtrII
(30%)
gtrII + gtrIV
(70%)
H1-Rha[I]
(Hybrid 2+4)
H1-GlcNAc
(Hybrid 2+4)
H1-Rha[I]
(2a)
H1-GlcNAc
(2a)
1.2521
0.4860
1.0000
0.4299
5.4
5.2
5.0
4.8
4.6
[ppm]
[rel]
0.4
0.3
0.2
0.1
-0.0

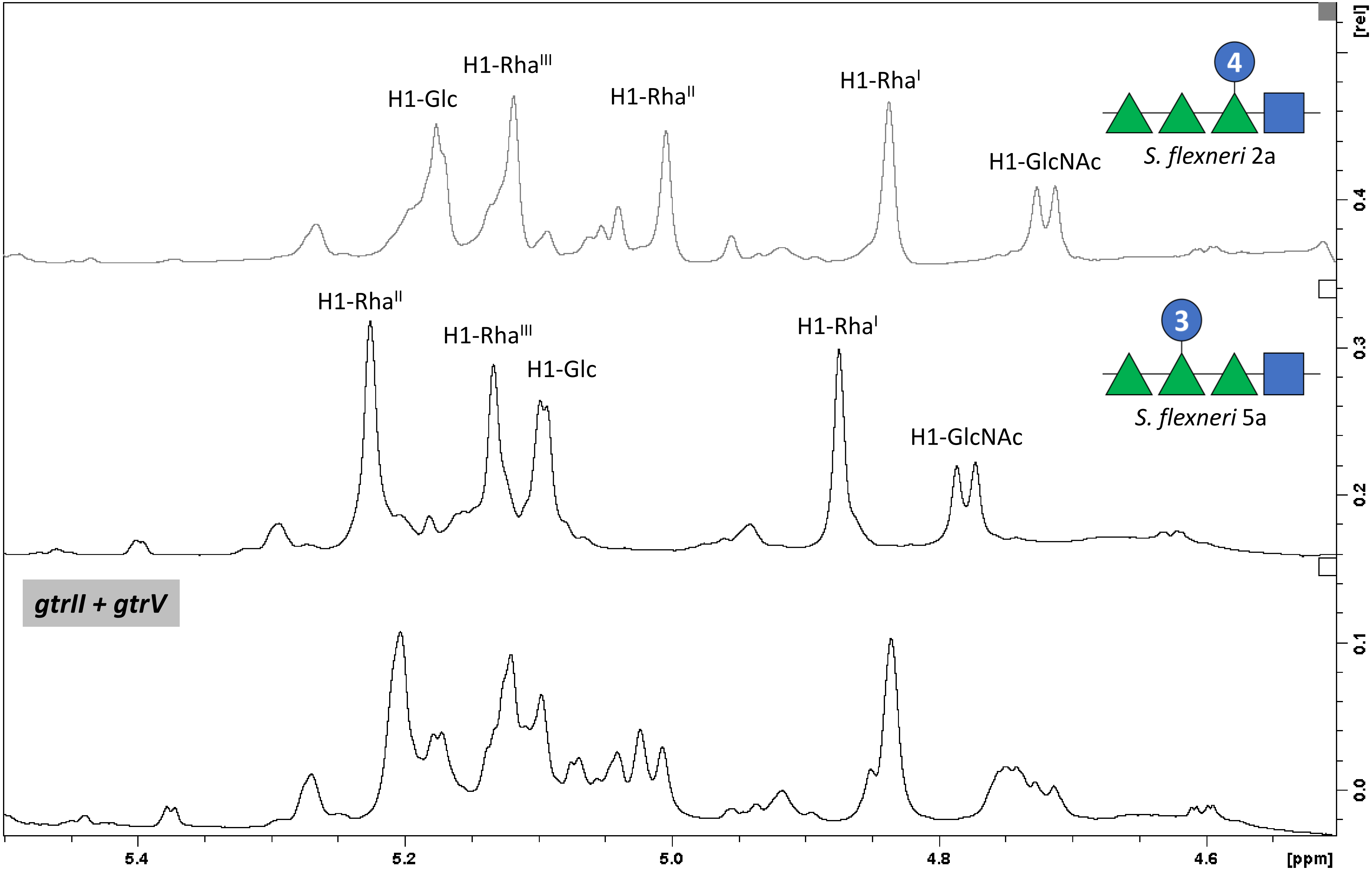
H1-Glc
H1-Rha^{III}
H1-Rha^{II}
H1-Rha^{I}
H1-GlcNAc
4
S. flexneri 2a
H1-Rha^{II}
H1-Rha^{III}
H1-Glc
H1-Rha^{I}
H1-GlcNAc
3
S. flexneri 5a
gtrII + gtrV
[rel]
0.4
0.3
0.2
0.1
0.0
5.4
5.2
5.0
4.8
4.6
[ppm]

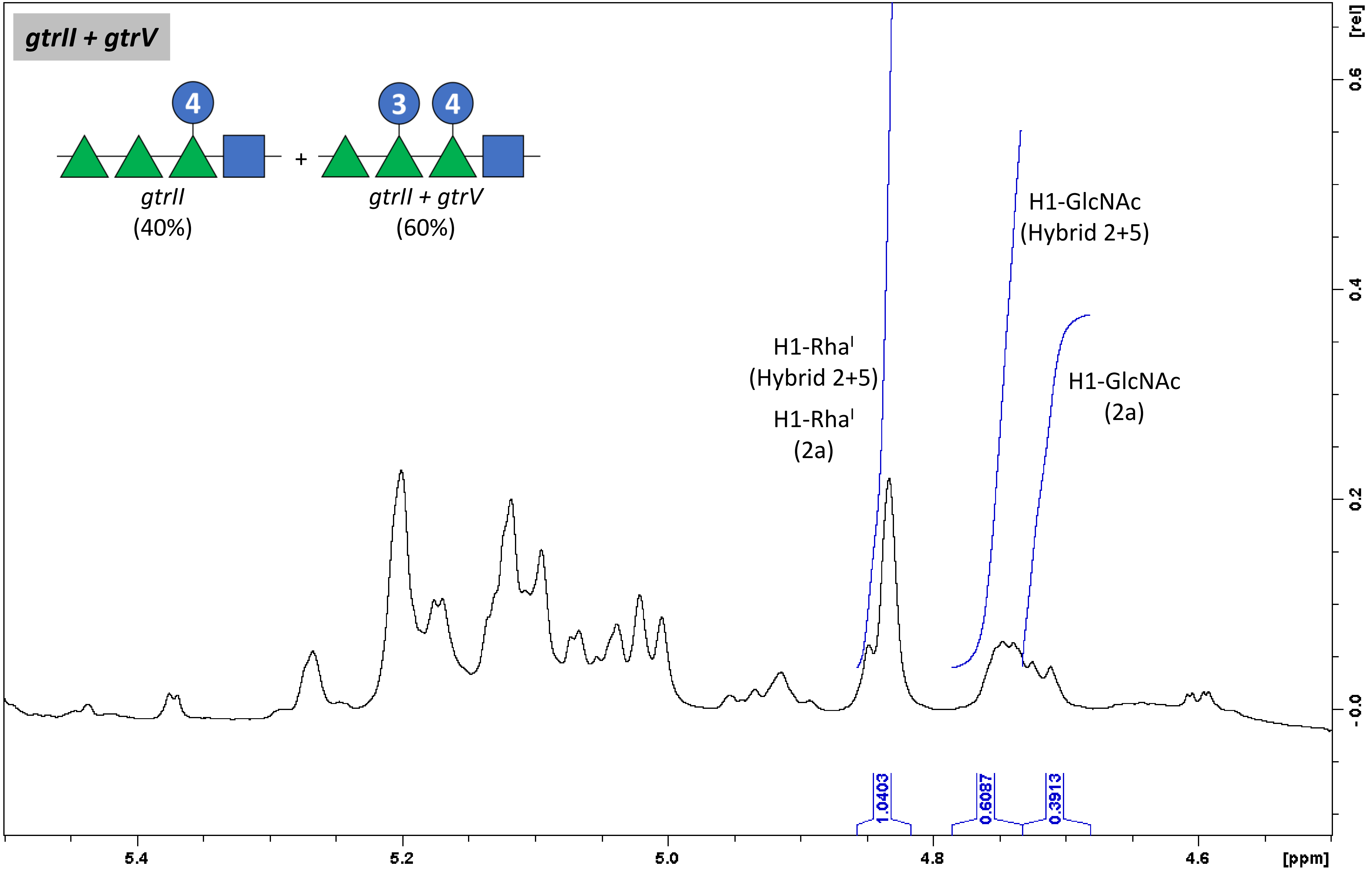
gtrII + gtrV
4
3
4
+
gtrII
(40%)
gtrII + gtrV
(60%)
H1-Rha[I]
(Hybrid 2+5)
H1-Rha[I]
(2a)
H1-GlcNAc
(Hybrid 2+5)
H1-GlcNAc
(2a)
1.0403
0.6087
0.3913
[rel]
0.6
0.4
0.2
-0.0
5.4
5.2
5.0
4.8
4.6
[ppm]

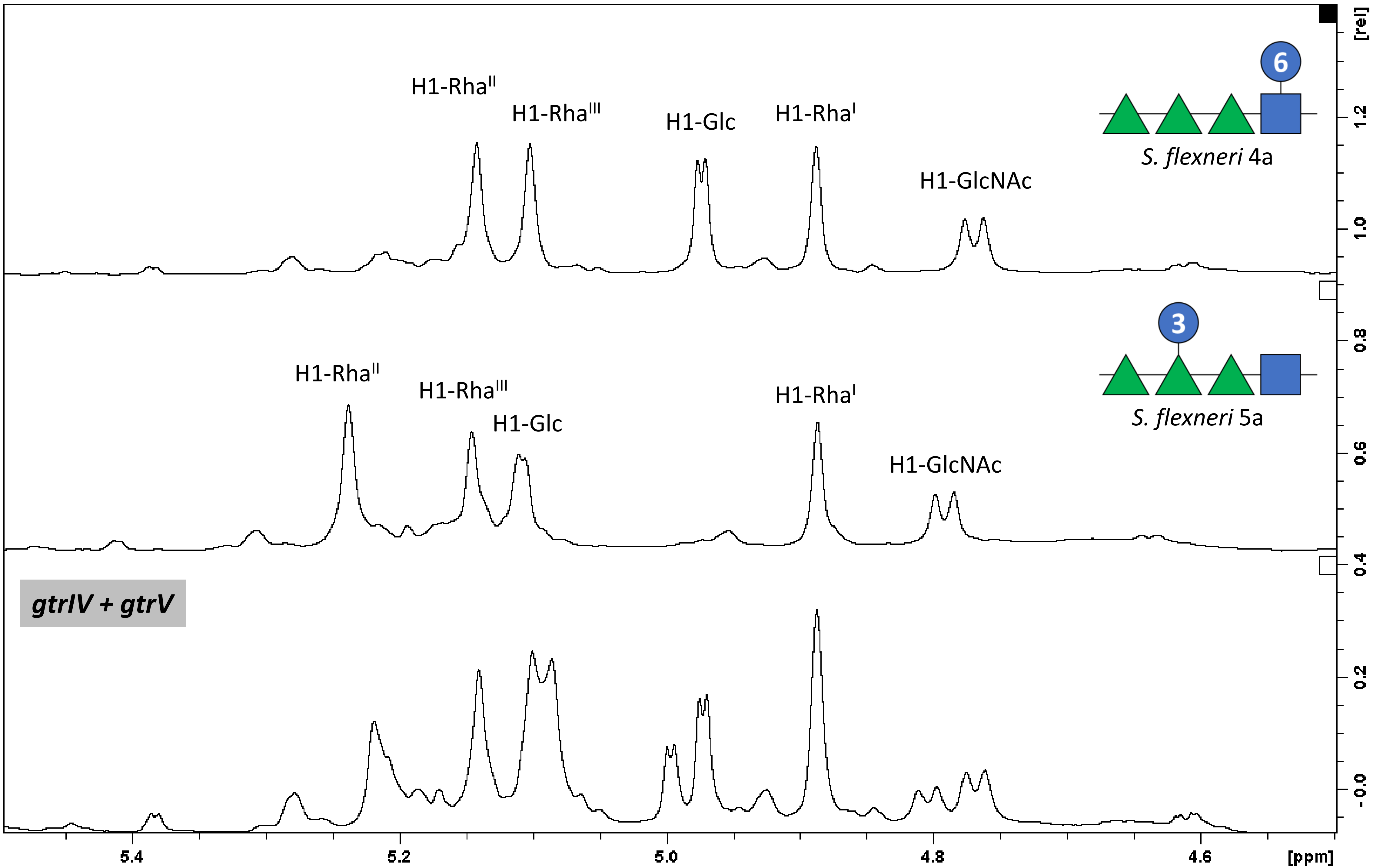

H1-Rha^{II}
H1-Rha^{III}
H1-Glc
H1-Rha^{I}
H1-GlcNAc
6
S. flexneri 4a
H1-Rha^{II}
H1-Rha^{III}
H1-Glc
H1-Rha^{I}
H1-GlcNAc
3
S. flexneri 5a
gtrIV + gtrV
5.4
5.2
5.0
4.8
4.6
[ppm]
[rel]
1.2
1.0
0.8
0.6
0.4
0.2
-0.0

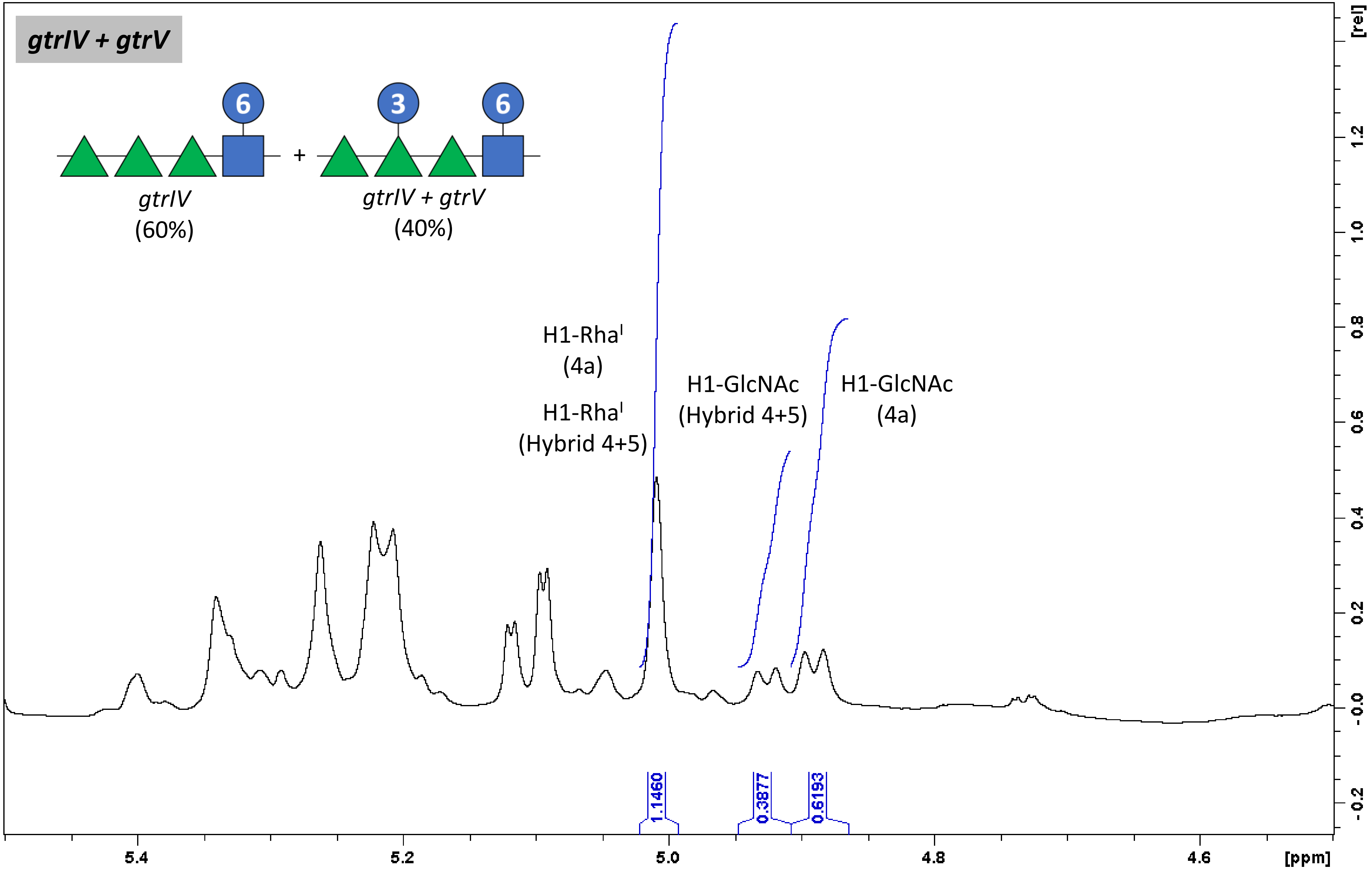
gtrIV + gtrV
6
3
6
+
gtrIV
(60%)
gtrIV + gtrV
(40%)
H1-Rha^I
(4a)
H1-Rha^I
(Hybrid 4+5)
H1-GlcNAc
(Hybrid 4+5)
H1-GlcNAc
(4a)
1.1460
0.3877
0.6193
5.4
5.2
5.0
4.8
4.6
[ppm]
[rel]
1.2
1.0
0.8
0.6
0.4
0.2
-0.0
-0.2

Incompatible enzymes

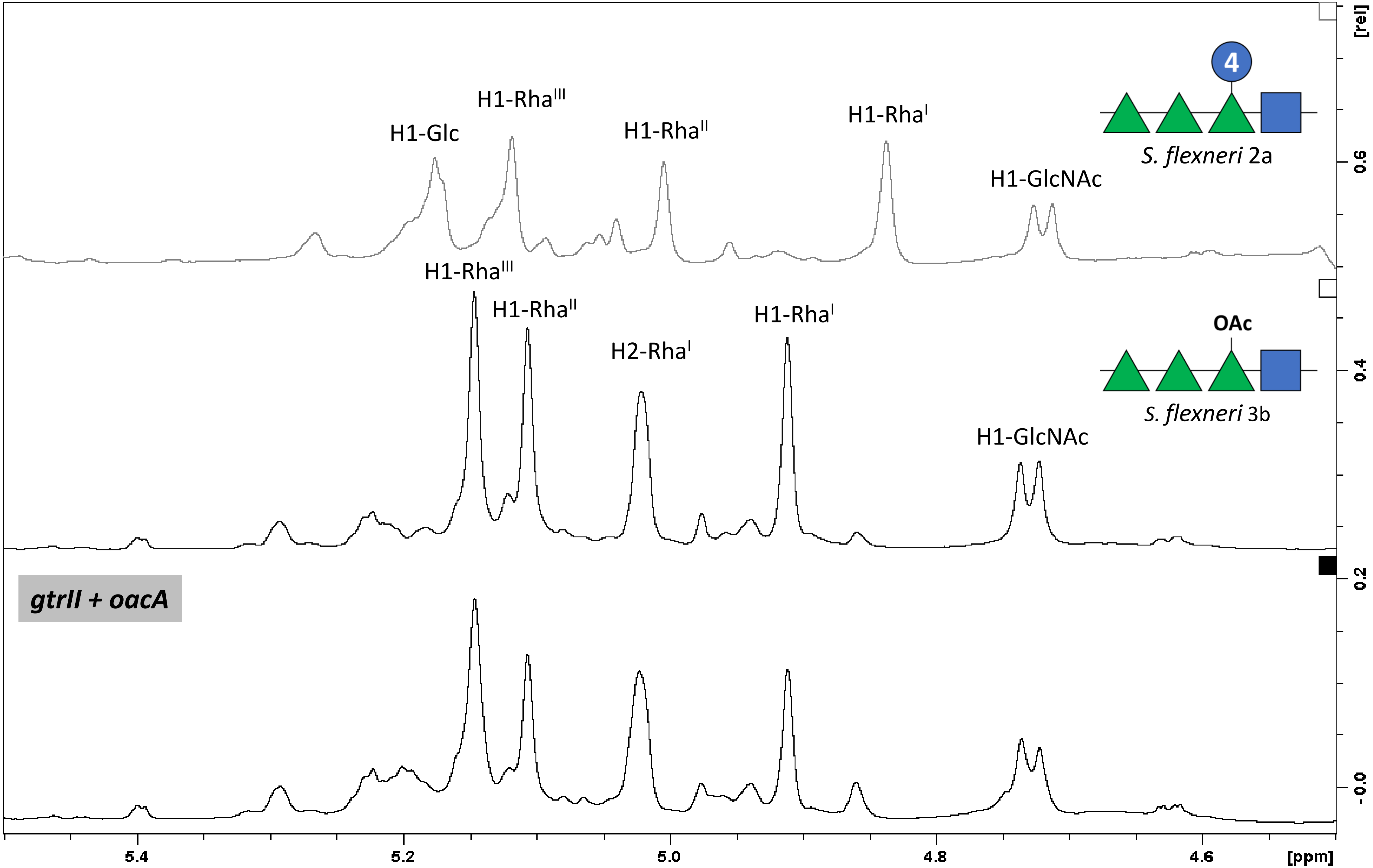

H1-Glc
H1-Rha^{III}
H1-Rha^{II}
H1-Rha^{I}
H1-GlcNAc
4
S. flexneri 2a
H1-Rha^{III}
H1-Rha^{II}
H2-Rha^{I}
H1-Rha^{I}
OAc
S. flexneri 3b
H1-GlcNAc
gtrII + oacA
5.4
5.2
5.0
4.8
4.6
[ppm]
[rel]
0.6
0.4
0.2
-0.0

References:

1. Perepelov, A. V.; Shekht, M. E.; Liu, B.; Shevelev, S. D.; Ledov, V. A.; Senchenkova, S. N.; L'Vov V, L.; Shashkov, A. S.; Feng, L.; Aparin, P. G.; Wang, L.; Knirel, Y. A., Shigella flexneri O-antigens revisited: final elucidation of the O-acetylation profiles and a survey of the O-antigen structure diversity. FEMS Immunol Med Microbiol **2012**, 66 (2), 201-10.
2. Perepelov, A. V.; L'Vov V, L.; Liu, B.; Senchenkova, S. N.; Shekht, M. E.; Shashkov, A. S.; Feng, L.; Aparin, P. G.; Wang, L.; Knirel, Y. A., A similarity in the O-acetylation pattern of the O-antigens of Shigellaflexneri types 1a, 1b, and 2a. *Carbohydr Res* **2009,** *344* (5), 687-92.
3. Shashkov, A. S.; Senchenkova, S. y. N.; Sun, Q.; Lan, R.; Wang, J.; Perepelov, A. V.; Knirel, Y. A.; Xu, J., Structure of the O-antigen of a novel Shigella flexneri serotype, 1d (I: 7,8). *Carbohydrate Research* **2013,** *373*, 93-96.
4. Jansson, P., Kenne, L., and Wehler, T., A 2D-1H-N.M.R. STUDY OF SOME Shigella flexneri O-POLYSACCHARIDES. *Carbohydrate Research* **1987,** *166*, 271-282.

a

Sample	Rha/Glc molar ratio (HPAEC-PAD)
Mixed 1+2 replicate 3	2.8
Mixed 1+2 replicate 2	2.5
Mixed 1+2 replicate 1	2.4

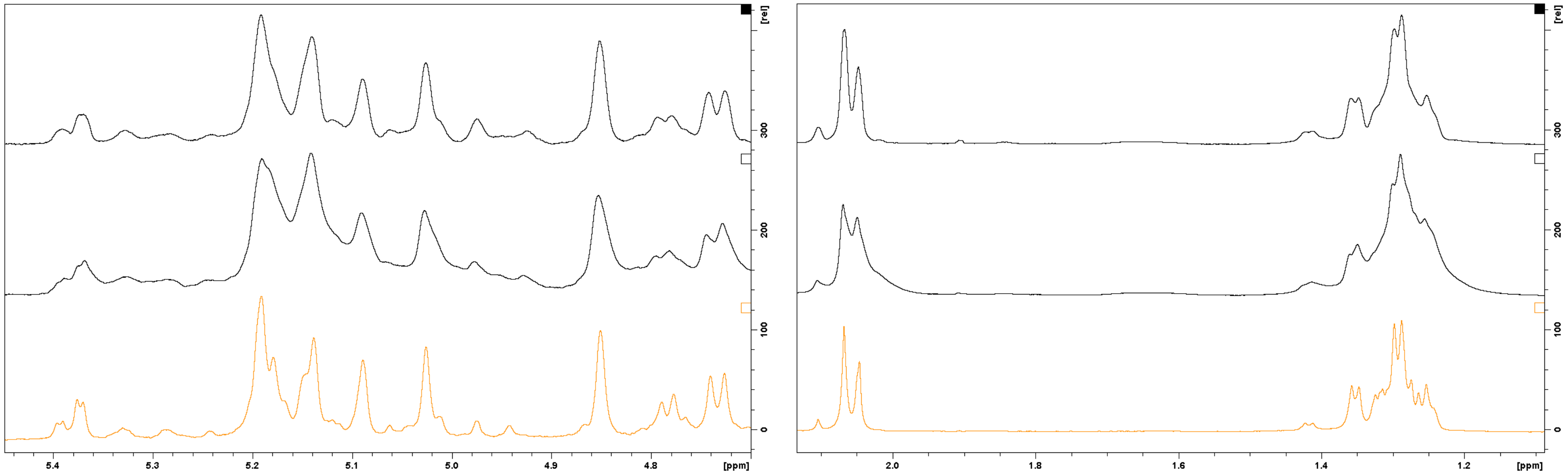

b

Sample	Rha/Glc molar ratio (HPAEC-PAD)
Hybrid 1+3 replicate 3	1.5
Hybrid 1+3 replicate 2	1.5
Hybrid 1+3 replicate 1	1.5

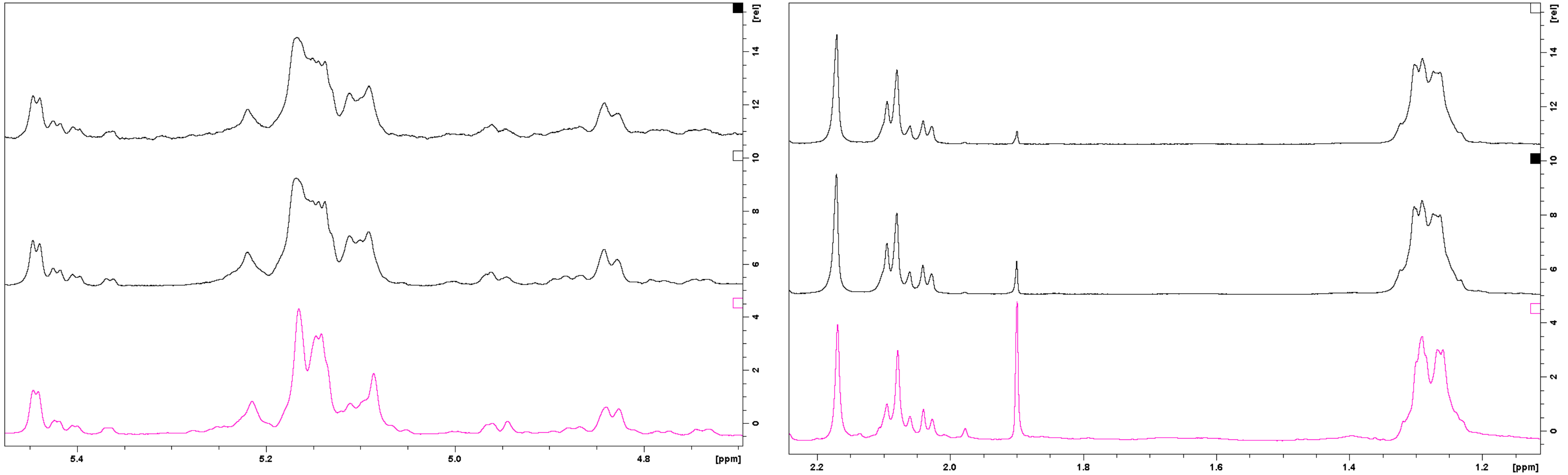

Supplement: Supplementary file 1 — Supplementary information [file 41541_2022_497_MOESM1_ESM.pdf]
